# Sex Differences in Behavioral and Psychopathological Trajectories From Late Childhood to Early Adolescence: Implications for Suicidality Risk

**DOI:** 10.1155/da/9546609

**Published:** 2025-12-01

**Authors:** Xiaoxia Duan, Yujie Tao, Mingjing Situ, Xinyi Yu, Di Jing, Pei Liu, Zhaozhi Yang, Yi Huang

**Affiliations:** ^1^Laboratory of Child and Adolescent Psychiatry, Mental Health Center and Psychiatric Laboratory, West China Hospital, Sichuan University, Chengdu, Sichuan 610041, China; ^2^Child Mental Health Center, West China Hospital, Sichuan University, Chengdu, Sichuan 610041, China; ^3^Mental Health Center, West China Hospital, Sichuan University, Chengdu, Sichuan 610041, China

**Keywords:** adolescence, attempted suicide, behaviors, psychopathology, suicidal ideation

## Abstract

**Background:**

Although the link between psychopathological and behavioral issues and suicidality is well-established, existing studies often focus on static timepoints, neglecting their dynamic nature and sex differences. This study investigates the trajectories of these symptoms from late childhood to early adolescence and their association with suicidality, while also examining variations by sex.

**Methods:**

We included 7849 unrelated children from the Adolescent Brain Cognitive Development (ABCD) cohort, with assessments conducted over a 3-year follow-up period. Caregiver-reported psychopathological and behavioral symptoms were assessed using the Child Behavior Checklist (CBCL) at baseline (ages 9–10) and during three subsequent annual follow-ups. Youth-reported suicidality was assessed at both baseline and the 3-year follow-up (ages 12–13) using the Kiddie Schedule for Affective Disorders and Schizophrenia (K-SADS).

**Results:**

Latent class growth analysis (LCGA) identified three trajectory groups for total problems, internalizing, and externalizing behaviors, and two trajectory groups for each dimension. All high symptom trajectory groups had a higher risk of suicidality compared to low symptom trajectory groups, with adjusted odds ratios (ORs) ranging from 1.35 to 3.15 (all *p*  < 0.05). Persistent high anxious/depressed symptoms showed the strongest association with suicidal ideation (SI; adjusted OR = 1.96, *p*  < 0.001), while persistent high attention problem was most strongly associated with suicide attempts (adjusted OR = 2.87, *p*  < 0.001). Persistently high anxious/depressed symptoms most strongly predicted suicide-related outcomes in girls (OR = 2.17, 95% confidence interval [CI]: 1.73–2.71), whereas high-increasing withdrawn/depressed symptoms showed the strongest association in boys (OR = 2.00, 95% CI: 1.53–2.60). Persistently high attention problems consistently and most strongly predicted suicide attempts in both sexes (boys: OR = 3.41, 95% CI: 1.52–7.65; girls: OR = 2.98, 95% CI: 1.72–5.14).

**Conclusions:**

Trajectories of psychopathological and behavioral symptoms from late childhood to early adolescence are modestly associated with suicidality during this critical transition. Withdrawn/depressed symptoms strongest predicted risk in boys, whereas anxious/depressed symptoms were most salient in girls. Attention problems consistently predicted suicide attempts across both sexes. These findings emphasize the need for ongoing monitoring and early intervention for at-risk youth to potentially reduce adolescent suicidality.

## 1. Introduction

Suicide serves as the most severe consequence of a broader mental health crisis affecting adolescents globally [1]. While adult suicide rates have declined due to various public health initiatives, adolescent suicide remains alarmingly high, constituting the second leading cause of death among youth aged 10–14 years worldwide [2]. In the United States, suicide mortality within this age group has significantly increased from 1.2 to 2.8 per 100,000 over the past two decades [3], continuing to be the leading cause of death, with an alarming rise in reports of suicidal thoughts and behaviors, particularly among those aged 12–15 [4]. A multinational meta-analysis identified suicide rates ranging from 0.93 to 6.04 per 100,000 during the childhood-to-adolescence transition [5]. Furthermore, a prospective longitudinal study tracking suicidal thoughts and behaviors from ages 3 to 17 noted a dramatic increase from ages 11 to 14 years [6]. Another investigation indicated that the prevalence of suicidal ideation (SI) among African American adolescents peaked between ages 12 and 13 in a cohort followed from ages 11 to 19 [7]. These alarming statistics underscore the urgent need for youth suicide prevention and the necessity for targeted early interventions during the critical developmental transition.

Children and adolescents represent a developmental phase characterized by significant neurobiological, cognitive, and psychosocial transformations, which significantly increase their vulnerability to mental health challenges [8]. The critical transition period serves a critical neurodevelopmental window, marked by extensive endocrine and neural reorganization [9, 10]. During this period, subclinical symptoms frequently progress into overt psychopathology [11]. A comprehensive meta-analysis encompassing 192 studies has identified 14.5 years as the peak age for the onset of mental disorders, revealing that nearly half of all mental disorders appear before age 18 [12]. These findings reinforce the urgency of recognizing and addressing mental health issues during adolescence [13]. Adolescence also presents unique vulnerabilities for suicide, influenced by an interplay of individual and environmental factors [ 14]. Identifying modifiable proximal risk factors for prevention is essential during this critical period characterized by rising suicide rates. Childhood psychopathological and behavioral symptoms serve as crucial and modifiable factors influencing the emergence of suicidal outcomes later in life [15]. For instance, research involving adolescents aged 10–17 has revealed that internalizing factors correlate with both suicidal thoughts and behaviors [16]. However, most studies focus on cross-sectional data, limiting our understanding of causality. Even longitudinal studies often focus solely on baseline symptoms, neglecting the dynamic nature of symptom trajectories across development. Furthermore, many investigations fail to account for sex-specific developmental pathways, despite emerging evidence indicating divergent psychopathological trajectories between girls and boys [17]. Such sex differences are particularly prominent during the peripubertal period, with females exhibiting greater vulnerability to internalizing disorders while males display elevated rates of externalizing behaviors [18]. Thus, examining potential sex-based differences in the influence of symptom trajectories on suicide risk is crucial for development of precisely targeted interventions.

The development of psychopathological and behavioral issues in adolescents is a complex and evolving process marked by notable diversity and change [19]. Longitudinal trajectories of these symptoms can vary widely, encompassing transient, recurrent, and chronic patterns [20]. Understanding these trajectories from childhood through adolescence is crucial, as it has important implications for suicide prevention during sensitive developmental stages [21]. Recent evidence indicates that high trajectories of childhood disruptive behaviors and anxiety can influence the relationship between early adversity and subsequent suicide attempts [22], while persistently high levels of depressive symptoms during adolescence correlate with adverse adult outcomes [23]. A study based on the Tokyo Teen Cohort examined the relationship between trajectories of psychopathological and behavioral symptoms and suicidal thoughts; however, this cohort was only followed up at ages 12 and 16, leaving a gap in understanding the critical transitional period from late childhood to early adolescence [24]. Consequently, the relationship between these trajectories across this transition and suicidal outcomes remains poorly defined, particularly from a sex-informed perspective. This highlighting a notable gap in research monitoring long-term symptom fluctuations in psychopathological and behavioral symptoms during this developmental stage, and investigates whether the associated risks differ meaningfully for boys and girls.

In the present study, we aim to investigate the developmental trajectories of psychopathological and behavioral symptoms from late childhood to early adolescence. We hypothesize that distinct symptom trajectories are significantly associated with an increased risk of SI and attempts during early adolescence. Additionally, we seek to assess potential variations in this risk based on sex. Our analysis focuses on over 7800 unrelated children from the Adolescent Brain Cognitive Development (ABCD) Study, who completed a baseline survey at ages 9–10, followed by an assessment at ages 12–13.

## 2. Methods

### 2.1. Study Participants

The ABCD Study is a nationwide, longitudinal cohort study initiated in 2016, designed to comprehensively examine adolescent brain development and behavior annually across the United States [25]. Funded by the National Institutes of Health, this initiative involves prominent experts in adolescent development and neuroscience to enhance our understanding of this critical period of life stage [ 26]. The study protocols received approval from the Institutional Review Board of the New York State Psychiatric Institute, ensuring ethical compliance, with informed consent secured from parents and verbal assent from participating minors.

This research utilized data from the Annual Curated Data Release 5.0 of the ABCD Study, focusing on a cohort of 11,868 children aged 9–10 years. To mitigate potential genetic confounding, one child was randomly selected from each family, resulting in a final pool of 9807 genetically unrelated participants. Participants with a history of traumatic brain injury (*n* = 147) or missing data on the Child Behavior Checklist (CBCL) (*n* = 1811) [27] were excluded, yielding an analytic sample of 7849 children (Figure S1).

### 2.2. Psychopathological and Behavioral Symptoms

Psychopathological and behavioral symptoms of children were assessed using the caregiver-reported CBCL [27], a widely recognized and empirically validated assessment tool comprising 113 items across eight syndrome scales (Table S1). Internalizing behaviors include the somatic complaints, anxious/depressed, and withdrawn/depressed scales, while externalizing behaviors encompass rule-breaking and aggressive behavior scales. Additional scales assess thought, attention, and social problems. This study analyzed total, internalizing, and externalizing scores, along with their subscales. To assess the longitudinal consistency of the CBCL scales, correlations between raw-score matrices for each psychopathological and behavioral symptom are presented in Figure S2, revealing modest correlations across the eight dimensions.

### 2.3. Suicidal Outcomes

Youth-reported suicidality was assessed at baseline and the 3-year follow-up using the computerized Kiddie Schedule for Affective Disorders and Schizophrenia (K-SADS), which captures both SI and attempts [28]. The baseline suicidal status was used as a covariate to account for pre-existing risk, whereas the 3-year follow-up assessment served as the primary outcome variable. SI was defined as thoughts, desires, or plans of self-harm with the intent to die. Participants were classified as experiencing SI if they endorsed characteristics such as passive suicidal thoughts, active but nonspecific ideation, specific method ideation, active ideation with intent, ideation with a formulated plan, or preparatory behaviors for imminent actions. Suicide attempt was explicitly defined as a specific episode of self-harming behavior undertaken with the conscious intention of ending one's life [ 29]. This definition encompassed actual, interrupted, or aborted attempts, capturing both current and historical occurrences.

### 2.4. Covariates

Informed by previous studies on suicide risk factors [30, 31], covariates included age, sex, social gender, race, grade, pubertal development, prodromal psychosis, bullying (victim, aggression, and non-bullying), cyberbullying, sleep disorder, body mass index (BMI), perinatal index, perceived discrimination, crystallized intelligence, fluid intelligence, sexual minority status, parental marital status, parental highest education, family conflict, family income, school environment, school involvement, school disengagement, and family history of suicide.

### 2.5. Statistical Analysis

#### 2.5.1. Descriptive Statistics

Data were analyzed from July to October 2024. Complete data for all CBCL scales and suicidal features were utilized in the study. Missing covariate values were addressed using multiple imputation with chained equations (*m* = 5 imputations, maxit = 20 iterations), implemented via the R “mice” package. Baseline characteristics were summarized using the first imputed dataset. Outliers in continuous variables were addressed using the winsorizing method [ 32]. Normally distributed quantitative variables were reported as means and standard deviations (SDs) and compared using Student's *t*-test, while non-normally distributed variables were presented as medians with the 25^th^ (P_25_) and 75^th^ (P_75_) percentiles and analyzed with the Mann–Whitney *U* test. Categorical variables were expressed as counts (*n*) and percentages (%) and assessed with the Chi-square test or Fisher's exact test as appropriate. All analyses were two-sided with significance set at *p*  < 0.05, conducted using R version 4.3.0.

#### 2.5.2. Latent Class Growth Analysis (LCGA)

LCGA was performed using Mplus software (version 8) [33] to identify distinct trajectory groups of psychopathological and behavioral symptoms. This method incorporates fixed effects of time and discrete random variables for latent classes, enabling nuanced exploration of high-resolution longitudinal data while assuming homogeneity within classes by fixing variance and covariance estimates for growth factors to zero. Trajectories of psychopathological and behavioral symptoms from late childhood to early adolescence were examined. Initially, a three-latent class model was fitted to determine the optimal trajectory shape by comparing linear, quadratic, and cubic models based on statistical fit indices and model stability assessments. The linear trajectory was identified as the most parsimonious and best-fitting solution. Subsequently, models with one to six trajectory groups characterized by linear slopes were evaluated, employing random starting values (*n* = 100) and a grid-search technique (with 20 iterations) to avoid local maxima. Optimal models were selected based on fit indices and statistical criteria, including lower Bayesian information criterion, sample size-adjusted Bayesian information criterion, and Akaike information criterion; higher entropy (> 0.8); a statistically significant Lo–Mendel–Rubin Likelihood Ratio Test (*p* < 0.05); an average posterior probability assignment exceeding 70%; and a minimum class size of at least 5% [23, 34, 35]. Reporting of the LCGA models followed the Guidelines for Reporting on Latent Trajectory Studies checklist [36] (Table S2). Trajectories were determined for the entire sample and separately for sex-stratified subgroups (male and female) across all CBCL scales. Detailed trajectory classifications and model fit parameters for each group are provided in Tables S3–S5.

#### 2.5.3. Logistic Regression Analysis

To investigate the relationship between suicidal features and trajectory groups of psychopathological and behavioral symptoms, logistic regression analyses were conducted. These analyses were performed on each imputed dataset, with results pooled according to Rubin's rules. Class membership based on CBCL scales was designated as the independent variable, with the lowest scoring class serving as the reference group. Suicidal features during the follow-up period were the dependent variable, with the participants who did not report any suicidal outcomes during this period classified as the control group. Odds ratios (ORs) with 95% confidence intervals (CIs) were calculated to assess the strength of associations. Three separate models were constructed for each CBCL scale: Model 0 was unadjusted, serving as the crude model; Model 1 included partial adjustments for baseline covariates such as age and sex; and Model 2 was fully adjusted for all relevant covariates, including baseline suicidality. Additionally, we assessed the association between sex-specific symptom trajectories and suicidal features exclusively within their respective sex groups through stratified analyses.

## 3. Results

### 3.1. Characteristics of Participants

The baseline characteristics and CBCL scale raw-scores of the study population are summarized in Table 1. The final cohort comprised 7849 unrelated children aged 9–10 years, with a mean age of 9.90 years (SD = 0.62), and 53.0% of participants were male. Following the baseline assessment, all participants completed three follow-up evaluations, with intervals of approximately 1.01 ± 0.01 years for the first follow-up, 1.10 ± 0.07 years for the second, and 0.90 ± 0.05 years for the third.

Table 1 presents the characteristics of participants categorized into three trajectories based on their total scores for psychopathological and behavioral symptoms as assessed by the CBCL. During the follow-up period, 2262 participants (28.82%) exhibited a persistent high level of CBCL total scores, 3823 participants (48.71%) exhibited a persistent middle level, and 1764 participants (22.47%) maintained persistent low levels. Notably, participants in the persistent high trajectory were significantly more likely to be male, have a higher BMI, experience greater family conflict, suffer from sleep disorders, be victims of cyberbullying and peer victimization, and report prodromal symptoms (all *p*  < 0.05). Furthermore, participants in the persistent high trajectory displayed the highest rates of suicidality, encompassing both SI and attempts (all *p*  < 0.05).

### 3.2. Trajectories of Psychopathological and Behavioral Symptoms

#### 3.2.1. Overall Sample Trajectories

**Figure 1:**
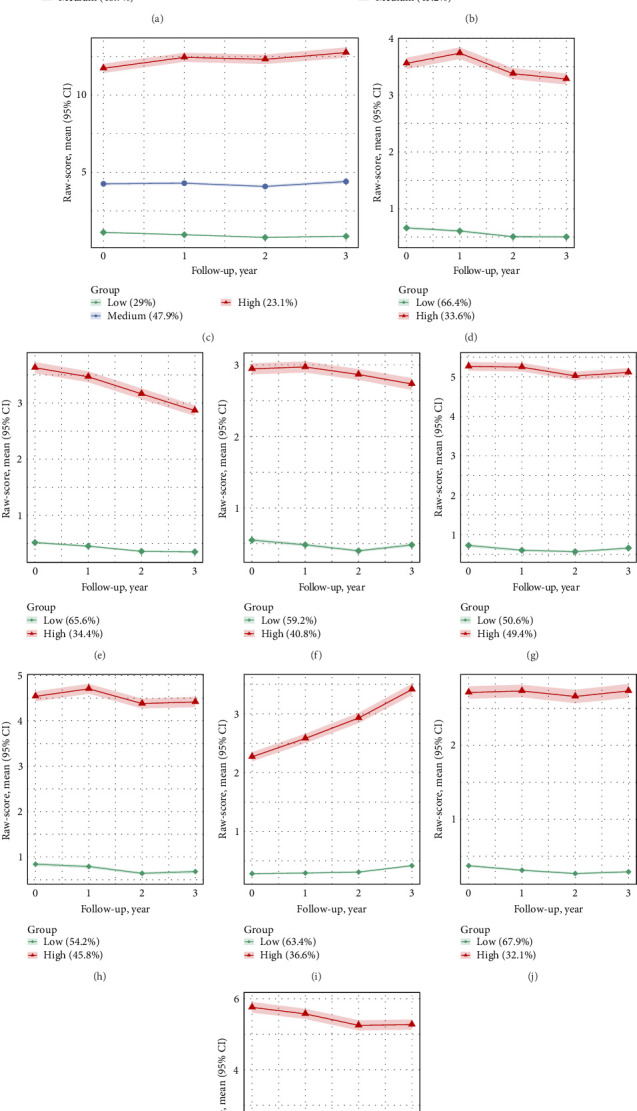
Trajectories of psychopathological and behavioral symptoms from late childhood to early adolescence, showing mean raw-scores and 95% CIs over the follow-up period. (A) Total problems. (B) Externalizing problems. (C) Internalizing problems. (D) Thought problems. (E) Social problems. (F) Somatic problems. (G) Attention problems. (H) Anxiety/depression problems. (I) Withdrawal/depression problems. (J) Rule-breaking behavior problems. (K) Aggressive behavior problems.

Based on the combination of statistical fit and interpretability, optimal models identified three trajectory groups for total problems, internalizing behaviors, and externalizing behaviors (Figure 1). The low total problems trajectory group included 1764 children (22.5%), while the persistent medium trajectory included 3823 children (48.7%), and the persistent high trajectory encompassed 2262 children (28.8%). Additionally, the persistent high internalizing behaviors trajectory group comprised 1817 children (23.1%), and the persistent high externalizing behaviors trajectory group included 1620 children (20.6%). For the eight CBCL subscales, a two-class solution provided clearer and more distinct trajectory separations. Among these, the attention problems subscale had the highest proportion of children in the sustained high trajectory group, accounting for 49.4%. This was followed by the aggressive problems subscale at 48.4%, and the anxious/depressed subscale at 45.8% in their respective high trajectory groups. The proportions of participants and the mean (SD) raw-scores for each CBCL subscale, stratified by trajectory group, are presented in Table S6.

#### 3.2.2. Sex Differences in Trajectories

**Figure 2:**
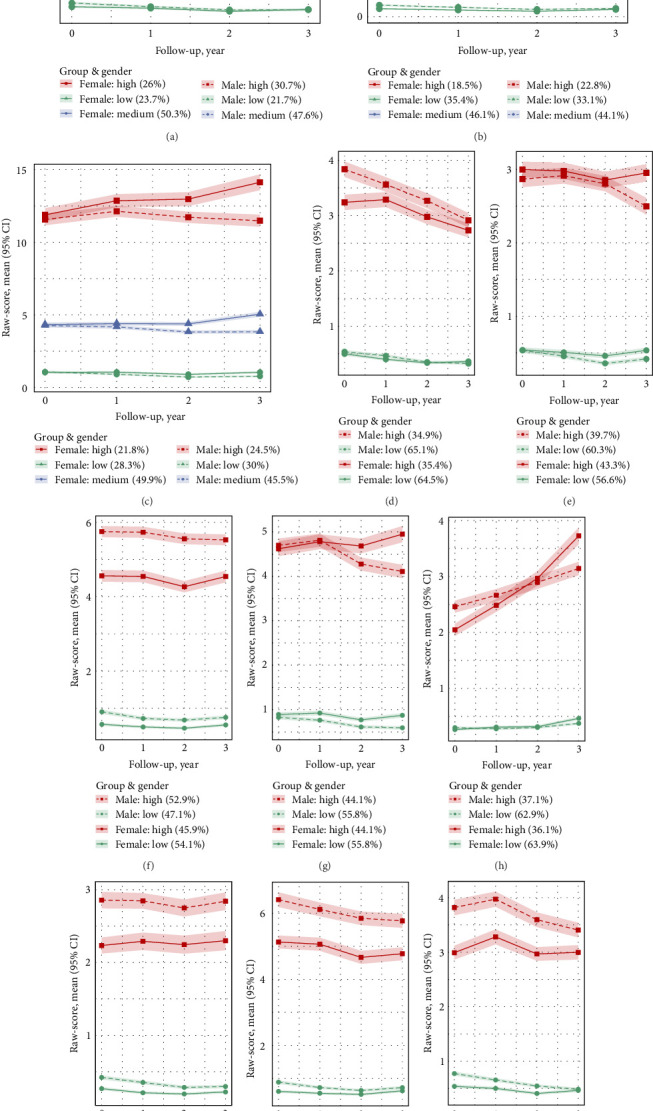
Trajectories of psychopathological and behavioral symptoms from late childhood to early adolescence stratified by sex, showing mean raw-scores and 95% CIs over the follow-up period. (A) Total problems. (B) Externalizing problems. (C) Internalizing problems. (D) Social problems. (E) Somatic problems. (F) Attention problems. (G) Anxiety/depression problems. (H) Withdrawal/depression problems. (I) Rule-breaking behavior problems. (J) Aggressive behavior problems. (K) Thought problems.

Figure 2 further illustrates the trajectory groups, displaying mean raw-scores and 95% CIs over the follow-up period, stratified by sex. Among girls, the trajectory groups closely resembled those of the overall sample. Notably, internalizing behaviors increased among girls in the high trajectory group. While somatic problems and anxious/depressed trajectories were similar for both sexes in the low-level groups, divergence was observed in the high-level groups, with girls holding steady and boys showing a declining trend. During this transition period, the trajectory level of the high withdrawal/depression group increased significantly for both genders, with girls exhibiting a faster rate of increase. The proportions of participants and the mean (SD) raw-scores for each CBCL subscale, stratified by trajectory group, time point, and sex, are provided in Tables S7 and S8.

### 3.3. Associations Between Trajectories and Suicidal Outcomes

#### 3.3.1. Associations in Overall Sample

**Figure 3:**
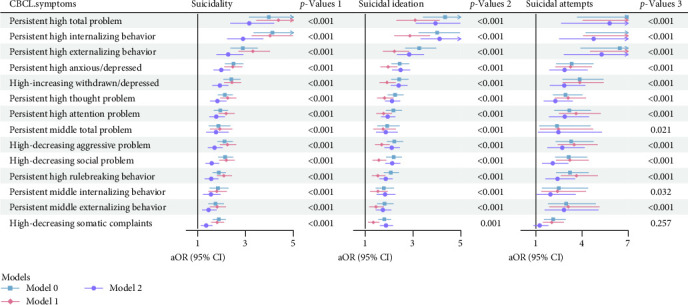
The association of associations of suicidality and psychopathological and behavioral symptoms trajectory group (Model 0 was unadjusted model; Model 1 was adjusted for age and sex; Model 2 was fully adjusted model, accounting for all relevant covariates; aOR refers to the adjusted odds ratio calculated from Model 2).

Compared to the low symptom trajectory groups, all high symptom trajectory groups showed higher risk of suicidality experience (Figure 3). The most pronounced difference was a 3.15 times greater risk in the persistent high total problems group (95% CI: 2.37–4.18). The persistent high internalizing behavior group had an adjusted OR of 2.89 (95% CI: 2.24–3.73), while the persistent high externalizing behavior group had an OR of 2.27 (95% CI: 1.79–2.89). Among the eight CBCL dimensions, children in higher symptom groups exhibited significantly increased risks of suicidality, with adjusted ORs ranging from 1.35 to 1.98 (all *p*  < 0.05). The group characterized by persistent high anxious/depressed symptoms showed the largest effect size, with an OR of 1.98 (95% CI: 1.67–2.35).

As shown in Figure 3, all symptoms were significantly associated with SI after adjusting for confounders, with adjusted ORs ranging from 1.34 to 3.09 (all *p*  < 0.05). With the exception of somatic symptoms, all symptoms were significantly associated with suicidal attempts. Notably, persistent high levels of total problems had the highest adjusted OR for suicidal attempts (OR = 5.79, 95% CI: 2.66–12.61), closely followed by persistent high levels of externalizing behavior (OR = 5.27, 95% CI: 2.83–9.81).

#### 3.3.2. Associations by Sex

**Figure 4:**
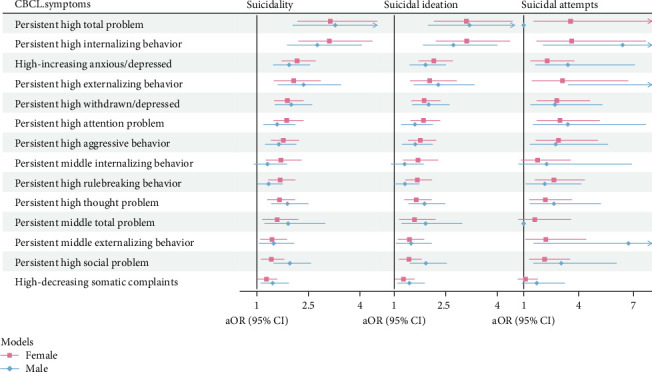
The association of associations of suicidality and psychopathological and behavioral symptoms trajectory group, stratified by sex (aOR refers to the adjusted odds ratio calculated from a fully adjusted model, accounting for all relevant covariates.).

High levels of CBCL total problems, internalizing behaviors, and externalizing behaviors were significantly associated with suicidality in both girls and boys (all *p*  < 0.05). Among the eight CBCL dimensions, persistent high levels of anxious/depressed symptom were significantly associated with all suicidal-outcomes both in boys and girls, showing the highest ORs for SI and attempts. The associations between the sex-specific symptom trajectories and suicidality are presented in Figure 4. Among the eight CBCL dimensions, the patterns of highest risk differed by sex: persistent anxious/depressed symptoms showed the highest risk of suicidality among girls (OR = 2.17, 95% CI: 1.73–2.71), whereas high-increasing withdrawn/depressed symptoms were associated with the greatest risk among boys (OR = 2.00, 95% CI: 1.53–2.60).

Among all CBCL dimensions, high trajectories of psychological and behavioral trajectories were consistently associated with increased risk of SI in both sexes, though with notable gender-specific variations in magnitude and profile (adjusted ORs ranged from 1.27 to 2.15 in girls and from 1.71 to 6.75 in boys, all *p*  < 0.05). Somatic symptoms constituted an exception, as their high trajectories were not significantly associated with suicide attempts in either sex (all *p* > 0.05). Notably, the pattern of strongest associations differed between boys and girls. For SI, persistent high anxious/depressed symptom was most strongly predictive in boys (adjusted OR = 2.00, 95% CI: 1.53–2.60), followed by the high-decreasing social problem (adjusted OR = 1.92, 95% CI: 2.46–2.52); whereas persistently high anxious/depressed symptoms showed the greatest risk among girls (adjusted OR = 2.15, 95% CI: 1.72–2.70). With respect to suicide attempts, persistently high attention problems emerged as the strongest predictor in both sexes, with adjusted ORs of 3.41 (95% CI: 1.52–7.65) in boys and 2.98 in girls (95% CI: 1.72–5.14) (Figure 4).

## 4. Discussions

This study represents the first prospective investigation into the long-term trajectories of psychopathological and behavioral symptoms from late childhood to early adolescence. Three trajectory groups for total problems, internalizing behaviors, and externalizing behaviors were identified, while two trajectory groups were observed for each CBCL dimension. These trajectories during this critical development transition are significantly associated with increased risks of both SI and attempts. Among these trajectories, persistent high anxious/depressed symptoms emerged as the strongest predictor of suicidality in girls, while in boys are high-increasing withdrawn/depressed symptoms. Additionally, persistent high attention problems were the strongest predictor for suicide attempts in both sexes.

We found that a persistent high trajectory of anxious/depressed symptoms emerged as the strongest predictor of suicidality. Previous studies have demonstrated these symptoms are significant predictors for SI among adolescents [37]. For instance, adolescent inpatients with higher depression scores are at an increased risk of new suicidal attempts [ 38]. Additionally, evidence from the Québec Longitudinal Study indicates that a profile characterized by high irritability and depressive/anxious mood is associated with suicide risk in children [ 39]. Our findings further validate this relationship from a prospective perspective, examining the developmental trajectory of anxiety/depressed symptoms from late childhood to early adolescence. This emphasizes that children with elevated levels of anxious/depressed symptoms face significantly heightened risk of suicidality during early adolescence. Consequently, there is a critical need for targeted preventive interventions aimed at addressing anxiety and depression during this pivotal developmental stage. By effectively identifying these at-risk individuals and implementing timely, evidence-based interventions, we can proactively mitigate the risk of suicide-related events and alleviate the associated societal health burden [40].

We also found a persistent high trajectory of social problems was significantly associated with increased risk of suicidality in early adolescence in both sexes, with the association being more pronounced in males. Our findings are consistent with a previous cross-sectional study that demonstrated a significant association between prolonged social withdrawal lasting 6 months or longer and increased risks of self-harm and suicidal behaviors [ 41]. Furthermore, another study found that avoidant problem is indirectly associated to SI through the severity of depressive symptom [42]. Research by Jia et al. [43] among patients with major depressive disorder illustrated that social withdrawal, a common clinical presentation, may increase the risk of suicide via emotional symptoms. Additionally, qualitative analyses suggest that various social warning signs, such as social withdrawal, significantly elevate the risk of suicidal behavior by creating conditions conductive to acute preparation and intent, which serve as critical indicators of imminent risk [44]. Using data from a prospective cohort, our study provides evidence of a longitudinal relationship between persistent high trajectory of social problems from late childhood to early adolescence and subsequent suicidality risk. Early identification of children facing social difficulties, especially in boys, coupled with the implementation of evidence-based treatments aimed at enhancing effective social problem-solving strategies—such as cognitive-behavioral therapy and acceptance and commitment therapy—can be instrumental in mitigating subsequent suicidal outcomes. We found individuals in groups with high trajectories of total problems, internalizing and externalizing behaviors exhibited increased risks of suicidality. Our findings align with existing literature that underscores the critical role of psychopathology in contributing to adverse mental health outcomes [45], such as suicidal attempts [24, 46]. We found in females, the highest-risk trajectories were persistent high anxious/depressed, high-increasing withdrawn/depressed, and persistent high attention problems. Among males, the most salient predictors included persistent high anxious/depressed, high-decreasing social problems, and high-increasing withdrawn/depressed. These results underscore the importance of implementing targeted intervention strategies tailored to address the specific psychopathological and behavioral issues faced by both male and female children in late childhood.

We identified a robust association between elevated attention problems and increased risk of suicidality in early adolescence. While evidence linking childhood attention problem to suicidality risk remains limited, existing studies suggest that these difficulties may contribute to be dysregulation and maladaptive coping strategies. For instance, elevated attention problems in childhood have been shown to predict a higher risk of cigaret smoking and delinquent behaviors in adolescence [47]. This pattern implies that deficits in attention and self-regulation may amplify susceptibility to impulsivity and risk-taking behaviors, which in turn could facilitate the emergence of SI and acts. Further studies are warranted to examine the specific mechanisms—such as impulsivity, emotional dysregulation, and social alienation—through which attention problems may influence pathways to suicide in developing youth. In addition, unlike a Chinese adolescent cohort study linking somatic symptoms and suicidal behavior [48], our study found no association between somatic complaints and suicidal attempts in children from ages 9 to 13. This discrepancy is likely due to differences in informant type, as the prior study used self-reports while ours relied on parent-reports. Other contributing factors may include cultural variations in symptom reporting, developmental stages, or follow-up duration. Further longitudinal research spanning broader developmental periods and incorporating different informants is warranted to clarify the association between somatic complaints and suicide risk.

In this study, we utilized LCGA to explore the developmental trajectories of psychopathological and behavioral symptoms from late childhood to early adolescence [49]. We have identified distinct trajectories of symptoms during the specific developmental phases and examined their relationship with suicidality, providing important insights for adolescent suicide prevention. However, it is important to exercise caution when interpreting the results due to the following limitations. First, similar to other longitudinal cohort studies, participant dropout and attrition are unavoidable issues. To minimize data loss, we employed multiple imputations to address the missing covariate information. Second, our reliance on children-reported K-SADS for suicidality may not fully align with parent reports, as previous studies indicate a low correlation and potential underreporting by parents [50]. Nonetheless, the use of the validated K-SADS tool allows for objective assessment of suicidality, particularly SI, effectively representing suicidal tendencies within this age group. Third, the limited number of data collection points may oversimplify complex developmental pathways. While capturing critical changes from late childhood to early adolescence, our analysis omits later adolescence, a period marked by significantly heightened suicide risk. Evidence shows a steep rise in suicide rates from pre-adolescence into young adulthood [ 51]. Continued longitudinal tracking within the ABCD cohort will be crucial to clarify how these trajectories vary and influence suicidal outcomes in later adolescence and young adulthood. Fourth, as the sample is US-based, findings may not generalize to other cultural contexts, given established global variations in youth suicide rates [51]. Finally, although our analysis identified clinically meaningful symptom patterns, the CBCL assesses observable behaviors rather than the underlying subjective dimension of mental pain—a profound form of psychological suffering considered central to suicidal behavior [52]. The trajectories we describe may, thus, reflect surface expressions of this deeper phenomenon; future studies should incorporate direct measures of mental pain to clarify its role in this mechanism.

In conclusion, this study identified three trajectory groups for total problems, internalizing behaviors, and externalizing behaviors within the overall sample, while two trajectory groups were discerned for each CBCL subscale. Trajectories marked by high symptom levels were consistently linked to increased risks of SI during adolescence. Notably, the persistent high trajectory of anxious/depressed symptoms from late childhood to early adolescence emerged as the strongest predictor of suicidality. Our findings reveal distinct, sex-specific pathways: while persistent high anxious/depressed symptoms constitute a major transdiagnostic risk factor in girls, high-increasing withdrawn/depressed symptoms represent the strongest predictor in boys. Attention problems consistently predicted suicide attempts across both sexes. This study suggests that dynamic psychiatric trajectories predict suicidality from late childhood to early adolescence, underscoring the need for early intervention and targeted support for at-risk youth.

## Figures and Tables

**Table 1:** Baseline characteristics of participants by trajectory group.

Characteristics	Total (*n* = 7849)	Persistent low (*n* = 1764)	Persistent medium (*n* = 3823)	Persistent high (*n* = 2262)	*p*-Value
Age, mean (SD), years	9.90 (0.62)	9.94 (0.62)	9.89 (0.61)	9.90 (0.62)	0.015
Sex (%)	—	—	—	—	<0.001
Male	4161 (53.0)	856 (48.5)	1973 (51.6)	1332 (58.9)	—
Female	3688 (47.0)	908 (51.5)	1850 (48.4)	930 (41.1)	—
Race (%)	—	—	—	—	<0.001
White	4256 (54.2)	915 (51.9)	2142 (56.0)	1199 (53.0)	—
Black	1010 (12.9)	264 (15.0)	443 (11.6)	303 (13.4)	—
Hispanic	1594 (20.3)	366 (20.7)	772 (20.2)	456 (20.2)	—
Asian	178 (2.3)	68 (3.9)	92 (2.4)	18 (0.8)	—
Other	811 (10.3)	151 (8.6)	374 (9.8)	286 (12.6)	—
Parental highest education (%)	—	—	—	—	<0.001
Less than HS diploma	315 (4.0)	95 (5.4)	134 (3.5)	86 (3.8)	—
HS diploma/GED	653 (8.3)	163 (9.2)	295 (7.7)	195 (8.6)	—
Some college or AA degree	1916 (24.4)	365 (20.7)	863 (22.6)	688 (30.5)	—
Bachelor's degree	2066 (26.3)	439 (24.9)	1035 (27.1)	592 (26.2)	—
Graduate and professional school	2893 (36.9)	702 (39.8)	1493 (39.1)	698 (30.9)	—
Parental marital status, married (%)	5505 (70.5)	1306 (74.5)	2761 (72.5)	1438 (64.0)	<0.001
Family income (%)	—	—	—	—	<0.001
$10,000 and greater	3192 (43.9)	775 (48.0)	1674 (46.7)	743 (35.8)	—
$50,000–100,000	2127 (29.2)	415 (25.7)	1060 (29.6)	652 (31.4)	—
Less than $5000	1953 (26.9)	423 (26.2)	849 (23.7)	681 (32.8)	—
Family conflict, mean (SD)	1.97 (1.93)	1.67 (1.76)	1.90 (1.90)	2.33 (2.05)	<0.001
Perceived discrimination, mean (SD)	1.17 (0.37)	1.13 (0.33)	1.15 (0.34)	1.24 (0.44)	<0.001
Sleep disorder, M (P_25_, P_75_)	35 (31, 40)	31 (29, 34)	34 (31, 38)	40 (35, 47)	<0.001
Physical activities days, M (P_25_, P_75_)	3 (2, 5)	4 (2, 5)	3 (2, 5)	3 (1, 5)	0.069
BMI, mean (SD), kg/m^2^	18.75 (4.14)	18.43 (3.78)	18.66 (4.00)	19.16 (4.60)	0.001
Grade, M (P_25_, P_75_)	4 (4, 5)	4 (4, 5)	4 (4, 5)	4 (4, 5)	0.003
Crystallized composite, M (P_25_, P_75_)	50 (44, 58)	51 (45, 58)	50 (44, 58)	49 (42, 56)	<0.001
Cognition fluid composite, M (P_25_, P_75_)	46 (38, 53)	47 (40, 55)	46 (39, 53)	44 (37, 52)	<0.001
School environment, M (P_25_, P_75_)	20 (18, 22)	21 (19, 22)	20 (18, 22)	20 (18, 22)	<0.001
School involvement, M (P_25_, P_75_)	13 (12, 15)	14 (12, 15)	13 (12, 15)	13 (11, 15)	<0.001
School disengagement, M (P_25_, P_75_)	4 (3, 5)	3 (2, 4)	4 (2, 5)	4 (3, 5)	<0.001
Perinatal index, mean (SD)	0.12 (0.83)	0.10 (0.82)	0.13 (0.83)	0.13 (0.84)	0.429
Prodromal symptoms, mean (SD)	1.27 (1.33)	1.06 (1.27)	1.21 (1.30)	1.54 (1.38)	<0.001
Sexual minority (%)	14 (0.2)	1 (0.1)	3 (0.1)	10 (0.4)	0.002
Cyberbullied (%)	704 (9.0)	106 (6.0)	301 (7.9)	297 (13.2)	<0.001
Bully (%)					<0.001
No	1915 (24.4)	563 (31.9)	917 (24.0)	435 (19.2)	—
Victim	5634 (71.8)	1129 (64.0)	2758 (72.1)	1747 (77.2)	—
Aggression	300 (3.8)	72 (4.1)	148 (3.9)	80 (3.5)	—
Parental history of suicide (%)	—	—	—	—	<0.001
G1^−^/G2^−^	6734 (89.8)	1621 (94.1)	3362 (91.9)	1751 (82.9)	—
G1^+^/G2^−^	350 (4.7)	63 (3.7)	151 (4.1)	136 (6.4)	—
G1^−^/G2^+^	343 (4.6)	32 (1.9)	127 (3.5)	184 (8.7)	—
G1^+^/G2^+^	68 (0.9)	7 (0.4)	20 (0.5)	41 (1.9)	—
Baseline history of suicidality (%)	677 (8.6)	67 (3.8)	290 (7.6)	320 (14.1)	<0.001
History of suicidal ideation (%)	666 (8.5)	66 (3.7)	286 (7.5)	314 (13.9)	<0.001
History of suicidal attempts (%)	87 (1.1)	5 (0.3)	25 (0.7)	57 (2.5)	<0.001

Abbreviations: BMI, body mass index; G1, generation 1 (grandparents); G2, generation 2 (parents); M, median; P25, the 25th percentile; P75, the 75th percentile; SD, standard deviation.

## Data Availability

The data that support the findings of this study are openly available in Adolescent Brain Cognitive Development cohort at https://abcdstudy.org/scientists/data-sharing/.

## Supporting Information

Supporting Information Figure S1. Illustrates the participant selection flowchart for the study. Table S1. Lists all items comprising each Child Behavior Checklist (CBCL) subscale. Figure S2. Displays heatmaps of Pearson correlation coefficients between raw-scores of CBCL scales across the four assessment periods used in latent class growth analysis. Table S2. Provides the completed Guidelines for Reporting on Latent Trajectory Studies (GRoLTS) checklist. Table S3. Presents model fit indices for latent class growth analysis including all participants. Table S4. Summarizes model fit indices for latent class growth analysis in male participants. Table S5. Details model fit indices for latent class growth analysis in female participants. Table S6. Reports means and standard deviations for CBCL subscales at all time points, stratified by trajectory class. Table S7. Provides means and standard deviations for CBCL subscales across all time points, by trajectory class in males. Table S8. Contains means and standard deviations for CBCL subscales across all time points, by trajectory class in females.
