# Supplementary material for: Sex Differences in Behavioral and Psychopathological Trajectories From Late Childhood to Early Adolescence: Implications for Suicidality Risk
Source: Depress Anxiety. 2025 Dec 1;2025:9546609. doi: 10.1155/da/9546609 (PMC12685420; doi:10.1155/da/9546609)
Supplement: Supporting Information — Figure S1. Illustrates the participant selection flowchart for the study. Table S1. Lists all items comprising each Child Behavior Checklist (CBCL) subscale. Figure S2. Displays heatmaps of Pearson correlation coefficients between raw-scores of CBCL scales across the four assessment periods used in latent class growth analysis. Table S2. Provides the completed Guidelines for Reporting on Latent Trajectory Studies (GRoLTS) checklist. Table S3. Presents model fit indices for latent class growth analysis including all participants. Table S4. Summarizes model fit indices for latent class growth analysis in male participants. Table S5. Details model fit indices for latent class growth analysis in female participants. Table S6. Reports means and standard deviations for CBCL subscales at all time points, stratified by trajectory class. Table S7. Provides means and standard deviations for CBCL subscales across all time points, by trajectory class in males. Table S8. Contains means and standard deviations for CBCL subscales across all time points, by trajectory class in females. [file 9546609.f1.docx]

**Supplementary Materials**

**Sex Differences in Behavioral and Psychopathological Trajectories from Late Childhood to Early Adolescence: Implications for Suicidality Risk**

**Table of contents**

| **Item** | **Page No.** |
| --- | --- |
| **Figure S1.** Study participants selection for the current study. | **2** |
| **Figure S2.** Heatmaps of Pearson correlation coefficients between Raw-scores of CBCL scales in four time-window used in latent class growth analysis. | **3** |
| **Table S1.** Items of Each Child Behavior Checklist (CBCL) Subscale. | 4-6 |
| **Table S2.** Report on Guidelines for Reporting on Latent Trajectory Studies (GRoLTS) | 7-8 |
| **Table S3.** Model Fit Indices for Latent Class Growth Analysis across all subjects. | 9-12 |
| **Table S4.** Model Fit Indices for Latent Class Growth Analysis in males. | 13-15 |
| **Table S5.** Model Fit Indices for Latent Class Growth Analysis in females. | 16-18 |
| **Table S6.** Means and Standard Deviations for CBCL subscales across all time points, separately for each class. | 19-20 |
| **Table S7.** Means and Standard Deviations for CBCL Subscales Across All Time Points, Separately for Each Class in Males. | 21-22 |
| **Table S8.** Means and Standard Deviations for CBCL Subscales Across All Time Points, Separately for Each Class in Females. | 23-24 |

**Supplementary figures**


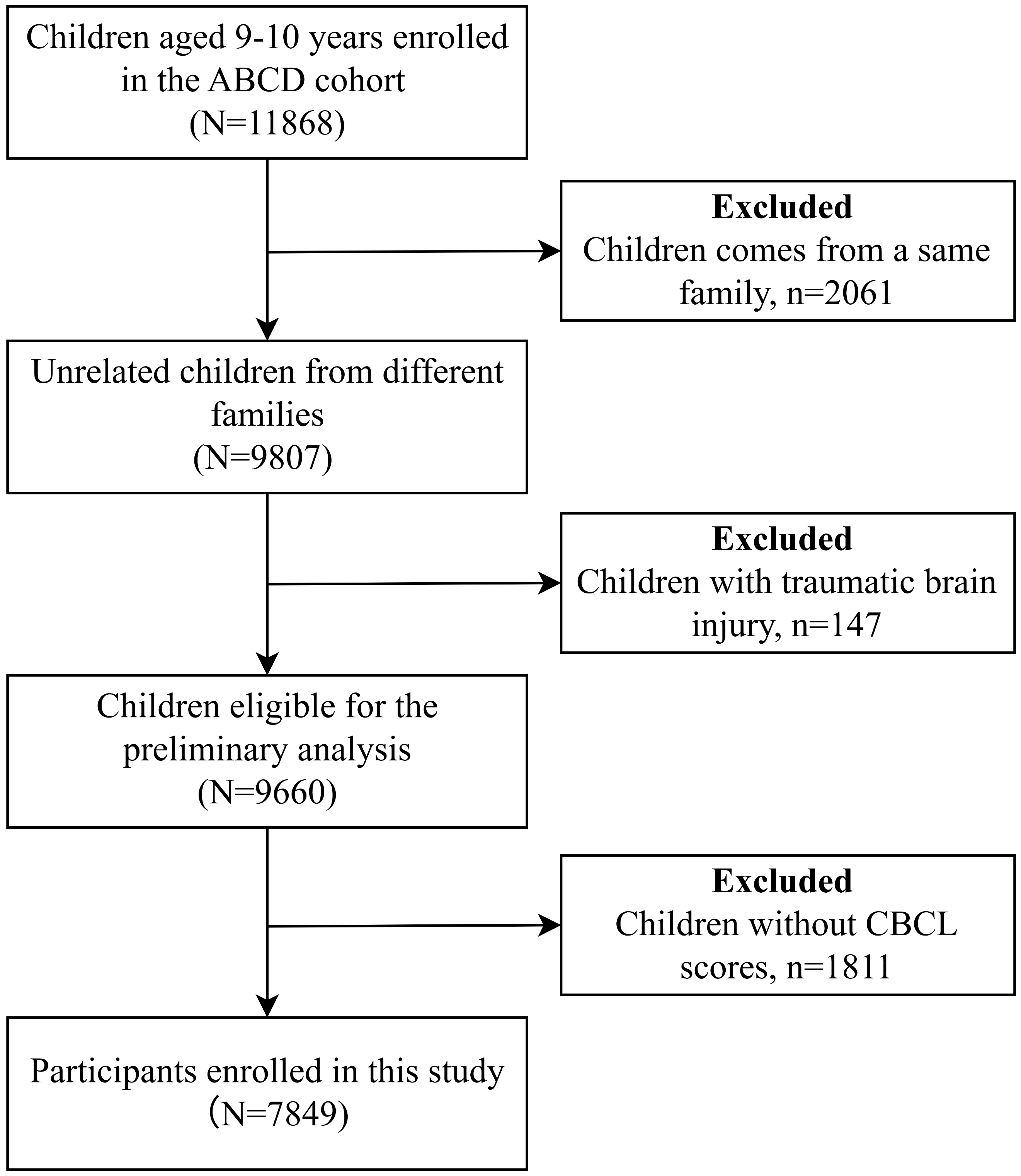


**Figure S1.** Study participants selection for the current study.


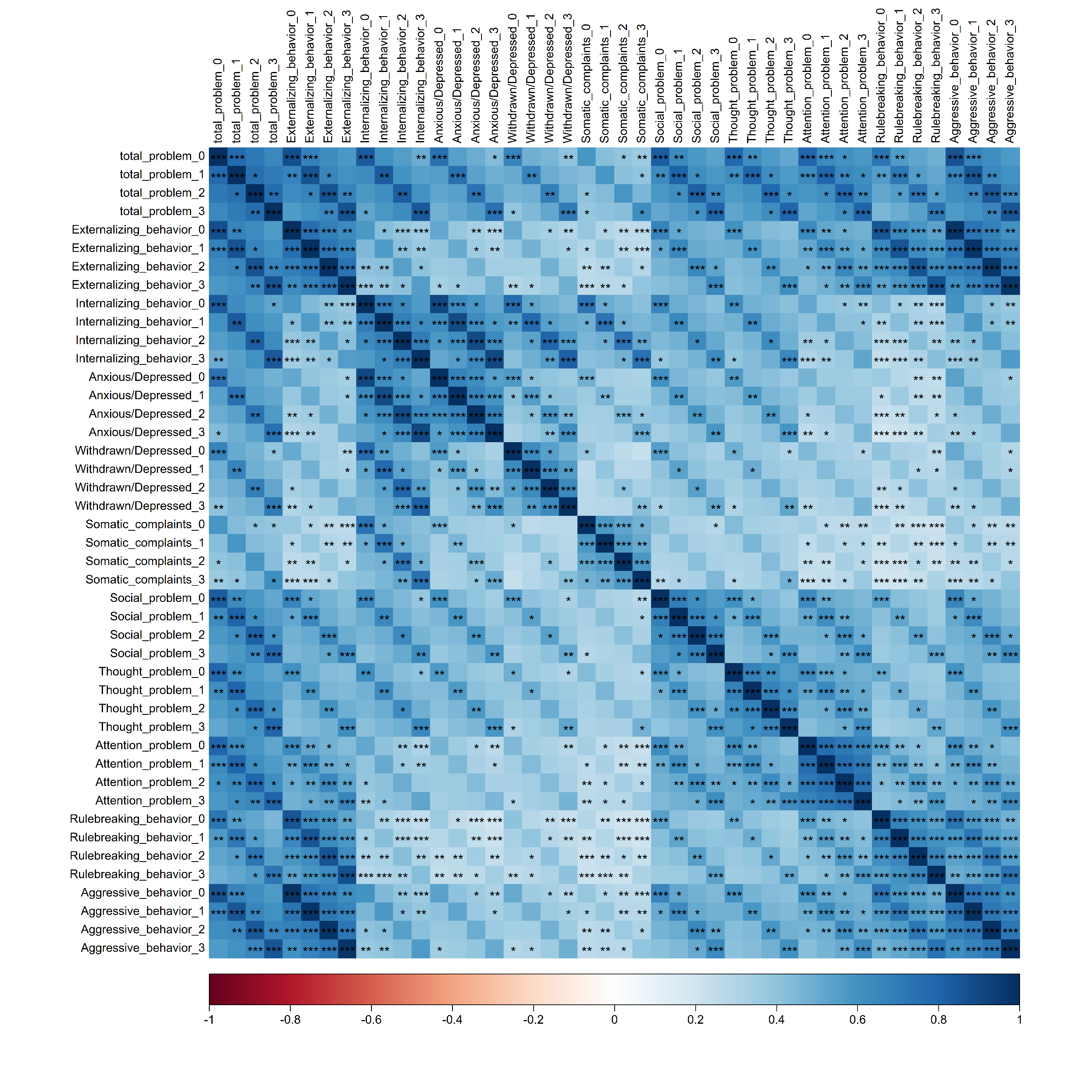


**Figure S2**. Heatmaps of Pearson correlation coefficients between Raw-scores of CBCL scales in four time-window used in latent class growth analysis. (Notes: **P*<0.05; ***P*<0.01; ****P*<0.001)

**Supplementary tables**

**Table S1.** Items of Each Child Behavior Checklist (CBCL) Subscale.

| **Subscale** | **Item** |
| --- | --- |
| Anxious/Depressed | 14. Cries a lot |
|  | 29. Fears certain animals, situations, or places, other than school |
|  | 30. Fears going to school |
|  | 31. Fears they might think or do something bad |
|  | 32. Feels they have to be perfect |
|  | 33. Feels or complains that no one loves them |
|  | 35. Feels worthless or inferior |
|  | 45. Nervous, high strung, or tense |
|  | 50. Too fearful or anxious |
|  | 52. Feels too guilty |
|  | 71. Self-conscious or easily embarrassed |
|  | 91. Talks about killing self |
|  | 112. Worries |
| Withdrawn/depressed | 5. There is very little they enjoy |
|  | 42. Would rather be alone than with others |
|  | 65. Refuses to talk |
|  | 69. Secretive, keeps things to self |
|  | 75. Too shy or timid |
|  | 102. Underactive, slow moving, or lacks energy |
|  | 103. Unhappy, sad, or depressed |
|  | 111. Withdrawn, doesn't get involved with others |
| Somatic complaints | 47. Nightmares |
|  | 49. Constipated, doesn't move bowels |
|  | 51. Feels dizzy or lightheaded |
|  | 54. Overtired without good reason |
|  | 56a. Aches or pains (not stomach or headaches) |
|  | 56b. Headaches |
|  | 56c. Nausea, feels sick |
|  | 56d. Problems with eyes (not if corrected by glasses) |
|  | 56e. Rashes or other skin problems |
|  | 56f. Stomachaches |
|  | 56g. Vomiting, throwing |
| Social problem | 11. Clings to adults or too dependent |
|  | 12. Complains of loneliness |
|  | 25. Doesn't get along with other kids |
|  | 27. Easily jealous |
|  | 34. Feels others are out to get them |
|  | 36. Gets hurt a lot, accident prone |
|  | 38. Gets teased a lot |
|  | 48. Not liked by other kids |
|  | 62. Poorly coordinated or clumsy |
|  | 64. Prefers being with younger kids |
|  | 79. Speech problem |
| Thought problem | 9. Can't get their mind off certain thoughts |
|  | 18. Deliberately harms self or attempts suicide |
|  | 40. Hears sound or voices that aren't there |
|  | 46. Nervous movements or twitching |
|  | 58. Picks nose, skin, or other parts of body |
|  | 59. Plays with own sex parts in public |
|  | 60. Plays with own sex parts too much |
|  | 66. Repeats certain acts over and over |
|  | 70. Sees things that aren't there |
|  | 76. Sleeps less than most kids |
|  | 83. Stores up too many things they don't need |
|  | 84. Strange behavior |
|  | 85. Strange ideas |
|  | 92. Talks or walks in sleep |
|  | 100. Trouble sleeping |
| Attention problem | 1. Acts too young for their age |
|  | 4. Fails to finish things they start |
|  | 8. Can't concentrate, can't pay attention for long |
|  | 10. Can't sit still, restless, or hyperactive |
|  | 13. Confused or seems to be in a fog |
|  | 17. Daydreams or gets lost in their thoughts |
|  | 41. Impulsive or acts without thinking |
|  | 61. Poor school work |
|  | 78. Inattentive or easily distracted |
|  | 80. Stares blankly |
| Rule-breaking behavior | 2. Drinks alcohol without parents' approval |
|  | 26. Doesn't seem to feel guilty after misbehaving |
|  | 28. Breaks rules at home, school or elsewhere |
|  | 39. Hangs around with others who get in trouble |
|  | 43. Lying or cheating |
|  | 63. Prefers being with older kids |
|  | 67. Runs away from home |
|  | 72. Sets fires |
|  | 73. Sexual problems |
|  | 81. Steals at home |
|  | 82. Steals outside the home |
|  | 90. Swearing or obscene language |
|  | 96. Thinks about sex too much |
|  | 99. Smokes, chews, or sniffs tobacco |
|  | 101. Truancy, skips school |
|  | 105. Uses drugs for non-medical purposes (don't include alcohol or tobacco) |
|  | 106. Vandalism |
| Aggressive behavior | 3. Argues a lot |
|  | 16. Cruelty, bullying, or meanness to others |
|  | 19. Demands a lot of attention |
|  | 20. Destroys their own things |
|  | 21. Destroys things belonging to their family or others |
|  | 22. Disobedient at home |
|  | 23. Disobedient at school |
|  | 37. Gets in many fights |
|  | 57. Physically attacks people |
|  | 68. Screams a lot |
|  | 86. Stubborn, sullen, or irritable |
|  | 87. Sudden changes in mood or feelings |
|  | 88. Sulks a lot |
|  | 89. Suspicious |
|  | 94. Teases a lot |
|  | 95. Temper tantrums or hot temper |
|  | 97. Threatens people |
|  | 104. Unusually loud |

**Table S2**. Report on Guidelines for Reporting on Latent Trajectory Studies (GRoLTS).

| **Checklist Item** | Reported? |
| --- | --- |
| **1.** Is the metric of time used in the statistical model reported? | Yes, the cohort initiated in 2016, and follow-up annually. |
| **2.** Is information presented about the mean and variance of time within a wave? | Yes, the means (SD) of time within a wave are mentioned in the results. |
| **3a.** Is the missing data mechanism reported? | Yes |
| **3b.** Is a description provided of what variables are related to attrition/missing data? | Yes, complete data for all CBCL scales and suicidality features were used, while missing data pertained to covariates. |
| **3c.** Is a description provided of how missing data in the analyses were dealt with? | Yes, missing covariate values imputed via maximum likelihood estimation. |
| **4.** Is information about the distribution of the observed variables included? | Yes, we presented the characteristics of participants in the Table 1. |
| **5.** Is the software mentioned? | Yes, we conducted the analyses using Mplus (version 8). |
| **6a.** Are alternative specifications of within-class heterogeneity considered (e.g., LGCA vs. LGMM) and clearly documented? If not, was sufficient justification provided as to eliminate certain specifications from consideration? | Yes, we used the LCGA model for count outcomes. |
| **6b.** Are alternative specifications of the between-class differences in variance-covariance matrix structure considered and clearly documented? If not, was sufficient justification provided as to eliminate certain specifications from consideration? | No |
| **7.** Are alternative shape/functional forms of the trajectories described? | Yes, models with linear slopes were evaluated. |
| **8.** If covariates have been used, can analyses still be replicated? | Not used. |
| **9.** Is information reported about the number of random start values and final iterations included? | Yes, a random starting value 100 and a grid-search technique was used (with 20 iterations) to avoid local maxima. |
| **10.** Are the model comparison (and selection) tools described from a statistical perspective? | Yes, optimal models were selected using fit indices and statistical criteria from the methods. |
| **11.** Are the total number of fitted models reported, including a one-class solution? | Yes, models with one to six trajectory groups were evaluated. |
| **12.** Are the number of cases per class reported for each model (absolute sample size, or proportion)? | Yes, the number of cases per class is reported in Supplementary Tables S6-8. |
| **13.** If classification of cases in a trajectory is the goal, is entropy reported? | Yes, we presented the entropy statics in Supplementary Table S3-5. |
| **14a.** Is a plot included with the estimated mean trajectories of the final solution? | Yes, the trajectory plots are shown in Figures 1 and 2. |
| **14b.** Are plots included with the estimated mean trajectories for each model? | No, we fitted a total of 150 models but chose not to display all of them due to space and clarity considerations. |
| **14c.** Is a plot included of the combination of estimated means of the final model and the observed individual trajectories split out for each latent class? | No, the plot is not included for the same reasons as 14b. |
| **15.** Are characteristics of the final class solution numerically described (i.e., means, SD/SE, n, CI, etc.)? | Yes, we reported means (SD) of each class in Supplementary Table S4. |
| **16.** Are the syntax files available (either in the appendix, supplementary materials, or from the authors)? | Available from authors on request. |

**Table S3.** Model Fit Indices for Latent Class Growth Analysis across all subjects.

| **No.** | **LL** | **df** | **AIC** | **BIC** | **aBIC** | **Entropy** | **LMR *P* value** | **(%) per class** | **APPA** |
| --- | --- | --- | --- | --- | --- | --- | --- | --- | --- |
| **Total problems** | | | | | | | | | |
| 1 | -121667 | 5 | 243346 | 243388 | 243369 | — | — | 100 | 100 |
| 2 | -115722 | 9 | 231461 | 231524 | 231495 | 0.827 | <0.001 | 53.0; 47.0 | 94.9; 94.9 |
| **3** | -113012 | 12 | 226047 | 226131 | 226093 | 0.843 | <0.001 | 48.7; 28.8; 22.5 | 93.2; 92.1; 93.0 |
| 4 | -111940 | 15 | 223910 | 224014 | 223967 | 0.820 | <0.001 | 37.9; 31.2; 12.1; 18.6 | 89.6; 88.8; 91.4; 89.3 |
| 5 | -111483 | 18 | 223003 | 223128 | 223071 | 0.794 | <0.001 | 9.2; 25.2; 33.6; 23.3; 8.6 | 83.3; 84.5; 86.3; 87.1; 90.2 |
| 6 | -111307 | 21 | 222656 | 222802 | 222735 | 0.775 | 0.006 | 4.0; 23.9; 21.8; 7.2; 12.3; 30.8 | 85.8; 82.2; 83.4; 81.2; 82.0; 84.1 |
| **Externalizing behaviors** | | | | | | | | | |
| 1 | -77806 | 6 | 155624 | 155666 | 155647 | — | — | 100 | 100 |
| 2 | -72218 | 9 | 144454 | 144516 | 144488 | 0.836 | <0.001 | 52.5; 47.5 | 95.7; 94.8 |
| **3** | -70035 | 12 | 140095 | 140178 | 140140 | 0.825 | <0.001 | 36.1; 20.6; 43.2 | 95.6; 90.0; 90.1 |
| 4 | -69295 | 15 | 138619 | 138724 | 138676 | 0.796 | <0.001 | 34.0; 27.1; 10.0; 28.9 | 84.6; 95.0; 86.4; 87.0 |
| 5 | -69073 | 18 | 138182 | 138308 | 138251 | 0.754 | 0.001 | 19.8; 29.4; 17.7; 5.2; 28.0 | 88.8; 80.8; 83.3; 88.5; 82.1 |
| 6 | -68901 | 21 | 137843 | 137990 | 137923 | 0.723 | 0.015 | 22.4; 4.8; 16.5; 17.2; 27.9; 11.3 | 93.8; 82.1; 82.4; 69.2; 83.2; 61.0 |
| **Internalizing behaviors** | | | | | | | | | |
| 1 | -85743 | 6 | 171498 | 171540 | 171521 | — | — | 100 | 100 |
| 2 | -80760 | 9 | 161539 | 161601 | 161573 | 0.805 | <0.001 | 51.1; 48.9 | 95.0; 93.6 |
| **3** | -78910 | 12 | 157844 | 157928 | 157890 | 0.799 | <0.001 | 47.9; 29.0; 23.1 | 91.0; 91.8; 89.1 |
| 4 | -78330 | 15 | 156690 | 156795 | 156747 | 0.768 | <0.001 | 37.9; 31.5; 20.6; 9.9 | 86.7; 85.3; 89.8;83.1 |
| 5 | -78153 | 18 | 156342 | 156467 | 156410 | 0.728 | <0.001 | 27.4; 11.0; 6.0; 22.4; 33.2 | 81.9; 80.4; 79.4; 81.6; 81.3 |
| 6 | -78014 | 21 | 156069 | 156215 | 156149 | 0.712 | 0.016 | 21.0; 22.2; 5.3; 32.3; 13.7; 5.5 | 81.2; 75.3; 51.9; 82.2; 87.1; 79.2 |
| **Thought problem** | | | | | | | | | |
| 1 | -53453 | 6 | 106919 | 106961 | 106942 | — | — | 100 | 100 |
| **2** | -48971 | 9 | 97961 | 98024 | 97995 | 0.801 | <0.001 | 66.4; 33.6 | 97.0; 89.2 |
| 3 | -47545 | 12 | 95114 | 95198 | 95159 | 0.784 | <0.001 | 12.4; 43.5; 44.1 | 87.5; 87.6; 93.0 |
| 4 | -47127 | 15 | 94284 | 94388 | 94341 | 0.753 | <0.001 | 43.3;31.9;5.0;19.9 | 85.7; 87.7; 85.9; 82.5 |
| 5 | -47058 | 18 | 94151 | 94277 | 94219 | 0.736 | 0.001 | 1.8; 28.8; 6.7; 42.6; 20.1 | 77.9; 86.5; 73.8; 85.5; 75.0 |
| 6 | -47027 | 21 | 94096 | 94243 | 94176 | 0.740 | 0.407 | 1.8; 0.6; 20.3; 28.8; 41.8; 6.7 | 79.1; 22.1; 75.5; 86.6; 84.9; 74.2 |
| **Social problem** | | | | | | | | | |
| 1 | -50623 | 6 | 101257 | 101299 | 101280 | — | — | 100 | 100 |
| **2** | -45910 | 9 | 91839 | 91901 | 91873 | 0.825 | <0.001 | 65.6; 34.4 | 97.6; 90.5 |
| 3 | -44624 | 12 | 89272 | 89356 | 89318 | 0.794 | <0.001 | 12.7; 46.0; 41.2 | 87.6; 91.3; 90.7 |
| 4 | -44309 | 15 | 88648 | 88752 | 88705 | 0.738 | <0.001 | 38.2; 35.8; 6.0; 20.0 | 84.2; 88.6; 84.7; 81.4 |
| 5 | -44234 | 18 | 88504 | 88629 | 88572 | 0.708 | <0.001 | 22.4; 9.1; 35.8; 30.4; 2.2 | 77.8; 76.3; 93.1; 71.8; 76.6 |
| 6 | -44188 | 21 | 88419 | 88565 | 88498 | 0.658 | 0.211 | 6.0; 2.2; 21.9; 35.8; 25.4; 8.7 | 36.4; 78.0; 78.2; 92.5; 68.0; 75.9 |
| **Attention problem** | | | | | | | | | |
| 1 | -69089 | 6 | 138189 | 138231 | 138212 | — | — | 100 | 100 |
| **2** | -62430 | 9 | 124878 | 124941 | 124913 | 0.873 | <0.001 | 50.6; 49.4 | 95.8; 96.8 |
| 3 | -60179 | 12 | 120382 | 120466 | 120428 | 0.845 | <0.001 | 24.3; 37.0; 38.7 | 93.3; 88.6; 97.1 |
| 4 | -59428 | 15 | 118887 | 118991 | 118943 | 0.811 | <0.001 | 27.4; 32.2; 27.8; 12.6 | 88.3; 87.2; 93.3; 88.9 |
| 5 | -59168 | 18 | 118372 | 118498 | 118440 | 0.766 | <0.001 | 27.8; 12.5; 27.5; 17.8; 14.4 | 93.8; 89.5; 89.5; 73.8; 69.3 |
| 6 | -58938 | 21 | 117918 | 118065 | 117998 | 0.741 | <0.001 | 14.3; 17.1; 24.9; 13.0; 23.3; 7.5 | 71.0; 82.9; 93.1; 67.8; 81.4; 85.5 |
| **Anxious depressed** | | | | | | | | | |
| 1 | -65244 | 6 | 130499 | 130541 | 130522 | — | — | 100 | 100 |
| **2** | -60412 | 9 | 120842 | 120904 | 120876 | 0.807 | <0.001 | 54.2; 45.8 | 94.8; 93.9 |
| 3 | -58830 | 12 | 117683 | 117767 | 117729 | 0.795 | <0.001 | 16.9; 38.2; 44.9 | 87.1; 94.3; 88.8 |
| 4 | -58366 | 15 | 116761 | 116866 | 116818 | 0.752 | <0.001 | 26.1; 37.5; 8.1; 28.3 | 90.5; 84.2; 83.9; 84.4 |
| 5 | -58270 | 18 | 116576 | 116701 | 116644 | 0.710 | 0.001 | 26.1; 32.3; 28.2; 8.0; 5.4 | 90.2; 80.3; 85.6; 84.7; 41.7 |
| 6 | -58163 | 21 | 116369 | 116515 | 116448 | 0.682 | 0.038 | 3.7; 22.0; 12.8; 26.8; 29.0; 5.7 | 79.3; 86.4; 75.3; 74.9; 80.7; 44.0 |
| **Withdrawn/depressed** | | | | | | | | | |
| 1 | -47423 | 6 | 94858 | 94900 | 94881 | — | — | 100 | 100 |
| **2** | -43340 | 9 | 86698 | 86760 | 86732 | 0.805 | <0.001 | 36.6; 63.4 | 91.1; 96.8 |
| 3 | -42381 | 12 | 84786 | 84870 | 84832 | 0.765 | <0.001 | 38.1; 49.0; 12.9 | 85.3; 94.9; 84.5 |
| 4 | -42170 | 15 | 84371 | 84475 | 84428 | 0.704 | <0.001 | 34.2; 39.1; 5.6; 21.2 | 77.1; 92.9; 79.1; 79.4 |
| 5 | -42041 | 18 | 84119 | 84244 | 84187 | 0.706 | <0.001 | 4.2; 19.6; 39.1; 32.2; 5.0 | 47.1; 81.2; 91.8; 76.8; 79.8 |
| 6 | -41997 | 21 | 84036 | 84182 | 84116 | 0.695 | 0.056 | 19.6; 7.9; 31.0; 4.1; 36.2; 1.1 | 74.8; 74.4; 72.6; 49.9; 90.3; 66.4 |
| **Somatic complaints** | | | | | | | | | |
| 1 | -52066 | 6 | 104143 | 104185 | 104166 | — | — | 100 | 100 |
| **2** | -48619 | 9 | 97257 | 97319 | 97291 | 0.749 | <0.001 | 40.8; 59.2 | 88.7; 95.4 |
| 3 | -47654 | 12 | 95332 | 95416 | 95378 | 0.742 | <0.001 | 48.6; 38.1; 13.4 | 89.5; 89.6; 82.4 |
| 4 | -47459 | 15 | 94948 | 95053 | 95005 | 0.694 | <0.001 | 4.9; 27.9; 44.4; 23.1 | 76.3; 83.2; 84.6; 78.0 |
| 5 | -47368 | 18 | 94772 | 94897 | 94840 | 0.671 | 0.010 | 4.5; 29.1; 22.2; 4.8; 39.4 | 77.7; 85.3; 77.9; 41.1; 81.0 |
| 6 | -47336 | 21 | 94715 | 94861 | 94794 | 0.605 | 0.279 | 22.6; 12.2; 29.1; 27.9; 5.5; 2.7 | 59.9; 69.2; 72.7; 91.0; 43.9; 72.8 |
| **Rule-breaking behavior** | | | | | | | | | |
| 1 | -44566 | 6 | 89144 | 89186 | 89167 | — | — | 100 | 100 |
| **2** | -40329 | 9 | 80676 | 80739 | 80711 | 0.815 | <0.001 | 67.9; 32.1 | 97.8; 89.1 |
| 3 | -39197 | 12 | 78419 | 78502 | 78464 | 0.794 | <0.001 | 36.9; 9.7; 53.3 | 86.3; 85.9; 95.6 |
| 4 | -38900 | 15 | 77831 | 77935 | 77888 | 0.751 | <0.001 | 15.9; 37.5; 43.3; 3.3 | 78.2; 83.6; 92.5; 81.8 |
| 5 | -38828 | 18 | 77693 | 77818 | 77761 | 0.705 | <0.001 | 40.0; 1.6; 31.8; 20.0; 6.7 | 92.0; 82.2; 70.9; 73.9; 72.8 |
| 6 | -38777 | 21 | 77596 | 77742 | 77675 | 0.668 | 0.005 | 6.6; 43.3; 1.6; 24.1; 20.7; 3.7 | 74.2; 96.3; 82.2; 62.8; 77.3; 30.1 |
| **Aggressive behavior** | | | | | | | | | |
| 1 | -69412 | 6 | 138836 | 138877 | 138858 | — | — | 100 | 100 |
| **2** | -63914 | 9 | 127845 | 127908 | 127879 | 0.834 | <0.001 | 51.6; 48.4 | 94.6; 94.2 |
| 3 | -61772 | 12 | 123567 | 123651 | 123613 | 0.829 | <0.001 | 34.2; 19.7; 46.1 | 91.9; 89.9; 93.3 |
| 4 | -61085 | 15 | 122200 | 122304 | 122257 | 0.799 | <0.001 | 26.6; 36.2; 28.6; 8.6 | 86.5; 87.5; 92.7; 87.2 |
| 5 | -60922 | 18 | 121879 | 122004 | 121947 | 0.745 | 0.005 | 27.5; 4.9; 15.6; 29.8; 22.2 | 80.7; 89.0; 82.7; 79.1; 91.0 |
| 6 | -60736 | 21 | 121513 | 121659 | 121593 | 0.721 | <0.001 | 15.0; 18.8; 25.7; 27.1; 8.4; 4.9 | 80.3; 71.9; 93.7; 82.4; 54.2; 85.0 |

**Note:** No.: Number of classes, df: degrees of freedom, LL: Log-likelihood, AIC: Akaike information criterion, BIC: Bayesian information criterion, aBIC: Simple size adjusted BIC, LMR: Lo-Mendell-Rubin, APPA: Average posterior probability of assignment.

**Table S4.** Model Fit Indices for Latent Class Growth Analysis in males.

| **No.** | **LL** | **df** | **AIC** | **BIC** | **aBIC** | **Entropy** | **LMR *P* value** | **(%) per class** | **APPA** |
| --- | --- | --- | --- | --- | --- | --- | --- | --- | --- |
| **Total problems** | | | | | | | | | |
| 1 | -65572 | 6 | 131155 | 131193 | 131174 | — | — | 100 | 100 |
| 2 | -62265 | 9 | 124548 | 124605 | 124577 | 0.838 | <0.001 | 53.1; 46.9 | 95.1; 95.5 |
| **3** | -60784 | 12 | 121592 | 121668 | 121630 | 0.847 | <0.001 | 30.1; 47.6; 21.7 | 92.4; 93.3; 93.0 |
| 4 | -60143 | 15 | 120316 | 120411 | 120363 | 0.828 | <0.001 | 13.0; 18.1; 31.8; 37.1 | 92.6; 89.3; 89.9; 89.6 |
| **Externalizing behaviors** | | | | | | | | | |
| 1 | -43110 | 6 | 86231 | 86269 | 86250 | — | — | 100 | 100 |
| 2 | -39955 | 9 | 79928 | 79985 | 79957 | 0.845 | <0.001 | 51.4; 48.6 | 95.5; 95.4 |
| **3** | -38742 | 12 | 77508 | 77584 | 77546 | 0.831 | <0.001 | 44.1; 22.9; 33.1 | 92.0; 90.7; 93.5 |
| 4 | -38292 | 15 | 76615 | 76710 | 76662 | 0.808 | <0.001 | 28.9; 35.8; 24.5; 10.8 | 87.6; 89.4; 90.9; 87.6 |
| **Internalizing behaviors** | | | | | | | | | |
| 1 | -44856 | 6 | 89725 | 89763 | 89744 | — | — | 100 | 100 |
| 2 | -42064 | 9 | 84146 | 84203 | 84175 | 0.819 | <0.001 | 49.0; 51.0 | 94.4; 95.1 |
| **3** | -41091 | 12 | 82206 | 82282 | 82244 | 0.800 | <0.001 | 45.5; 30.0; 24.5 | 90.1; 92.5; 89.8 |
| 4 | -40760 | 15 | 81550 | 81645 | 81597 | 0.780 | <0.001 | 38.4; 21.9; 9.0; 30.6 | 87.5; 90.4; 82.4; 86.0 |
| **Thought problem** | | | | | | | | | |
| 1 | -29425 | 6 | 58862 | 58900 | 58881 | — | — | 100 | 100 |
| **2** | -26857 | 9 | 53732 | 53789 | 53760 | 0.814 | <0.001 | 65.2; 34.8 | 95.9; 91.9 |
| 3 | -25999 | 12 | 52022 | 52098 | 52060 | 0.795 | <0.001 | 13.3; 43.2; 43.5 | 88.2; 94.2; 88.0 |
| 4 | -25732 | 15 | 51493 | 51588 | 51541 | 0.767 | <0.001 | 20.1; 5.9; 31.4; 42.6 | 83.2; 88.5; 89.3; 85.9 |
| **Social problem** | | | | | | | | | |
| 1 | -27255 | 6 | 54523 | 54561 | 54542 | — | — | 100 | 100 |
| **2** | -24594 | 9 | 49206 | 49263 | 49235 | 0.836 | <0.001 | 34.9; 65.1 | 92.1; 97.3 |
| 3 | -23826 | 12 | 47677 | 47753 | 47714 | 0.806 | <0.001 | 47.0; 13.6; 39.5 | 92.7; 89.5; 89.8 |
| 4 | -23660 | 15 | 47349 | 47444 | 47397 | 0.756 | 0.078 | 18.4; 38.9; 36.7; 5.9 | 81.5; 86.0; 87.3; 83.9 |
| **Attention problem** | | | | | | | | | |
| 1 | -38949 | 6 | 77909 | 77947 | 77928 | — | — | 100 | 100 |
| **2** | -35220 | 9 | 70458 | 70515 | 70487 | 0.881 | <0.001 | 52.9; 47.1 | 97.2; 95.8 |
| 3 | -33910 | 12 | 67845 | 67921 | 67883 | 0.851 | <0.001 | 27.3; 32.7; 40.0 | 93.4; 93.6; 92.6 |
| 4 | -33458 | 15 | 66946 | 67041 | 66993 | 0.819 | <0.001 | 32.5; 23.2; 29.1; 15.2 | 90.2; 90.1; 88.5; 90.2 |
| **Anxious/depressed** | | | | | | | | | |
| 1 | -33942 | 6 | 67897 | 67935 | 67916 | — | — | 100 | 100 |
| **2** | -31220 | 9 | 62457 | 62514 | 62486 | 0.823 | <0.001 | 44.1; 55.9 | 93.1; 96.3 |
| 3 | -30408 | 12 | 60840 | 60916 | 60877 | 0.792 | <0.001 | 18.8; 37.9; 43.3 | 88.5; 92.7; 89.2 |
| 4 | -30136 | 15 | 60303 | 60398 | 60350 | 0.767 | <0.001 | 28.3; 37.1; 7.2; 27.3 | 91.0; 84.9; 83.6; 85.9 |
| **Withdrawn/depressed** | | | | | | | | | |
| 1 | -25219 | 6 | 50451 | 50489 | 50469 | — | — | 100 | 100 |
| **2** | -22913 | 9 | 45845 | 45902 | 45873 | 0.814 | <0.001 | 62.9; 37.1 | 97.5; 90.5 |
| 3 | -22359 | 12 | 44742 | 44818 | 44779 | 0.780 | <0.001 | 12.8; 49.5; 37.8 | 84.7; 95.4; 86.3 |
| 4 | -22232 | 15 | 44495 | 44590 | 44542 | 0.728 | <0.001 | 34.6; 39.5; 4.7; 21.2 | 80.1; 92.3; 77.5; 81.9 |
| **Somatic complaints** | | | | | | | | | |
| 1 | -26714 | 6 | 53440 | 53478 | 53459 | — | — | 100 | 100 |
| **2** | -24905 | 9 | 49829 | 49886 | 49857 | 0.750 | <0.001 | 39.7; 60.3 | 89.7; 94.4 |
| 3 | -24406 | 12 | 48837 | 48913 | 48875 | 0.746 | <0.001 | 47.4; 41.3; 11.3 | 89.1; 90.1; 81.6 |
| 4 | -24288 | 15 | 48606 | 48701 | 48653 | 0.698 | <0.001 | 22.1; 43.6; 30.2; 4.1 | 77.9; 83.6; 85.9; 78.0 |
| **Rule-breaking behavior** | | | | | | | | | |
| 1 | -25673 | 6 | 51357 | 51395 | 51376 | — | — | 100 | 100 |
| **2** | -23255 | 9 | 46527 | 46584 | 46556 | 0.819 | <0.001 | 62.6; 37.4 | 95.8; 93.5 |
| 3 | -22585 | 12 | 45194 | 45270 | 45232 | 0.793 | <0.001 | 11.4; 47.9; 40.1 | 85.7; 93.3; 89.6 |
| 4 | -22428 | 15 | 44885 | 44980 | 44932 | 0.754 | <0.001 | 3.9; 40.4; 18.1; 37.5 | 80.1; 86.5; 81.4; 88.4 |
| **Aggressive behavior** | | | | | | | | | |
| 1 | -38298 | 6 | 76608 | 76646 | 76627 | — | — | 100 | 100 |
| **2** | -35220 | 9 | 70458 | 70515 | 70486 | 0.843 | <0.001 | 51.8; 48.2 | 95.8; 95.2 |
| 3 | -34024 | 12 | 68073 | 68149 | 68110 | 0.832 | <0.001 | 43.4; 22.2; 34.4 | 90.5; 91.2; 94.8 |
| 4 | -33591 | 15 | 67211 | 67306 | 67259 | 0.811 | <0.001 | 26.9; 9.5; 36.3; 27.3 | 91.5; 88.1; 89.3; 87.2 |

**Note:** No.: Number of classes, df: degrees of freedom, LL: Log-likelihood, AIC: Akaike information criterion, BIC: Bayesian information criterion, aBIC: Simple size adjusted BIC, LMR: Lo-Mendell-Rubin, APPA: Average posterior probability of assignment.

**Table S5.** Model Fit Indices for Latent Class Growth Analysis in females.

| **No.** | **LL** | **df** | **AIC** | **BIC** | **aBIC** | **Entropy** | **LMR *P* value** | **(%) per class** | **APPA** |
| --- | --- | --- | --- | --- | --- | --- | --- | --- | --- |
| **Total problems** | | | | | | | | | |
| 1 | -55993 | 6 | 111998 | 112035 | 112016 | — | — | 100 | 100 |
| 2 | -53366 | 9 | 106749 | 106805 | 106777 | 0.817 | <0.001 | 52.8; 47.2 | 94.9; 94.5 |
| **3** | -52155 | 12 | 104334 | 104408 | 104370 | 0.836 | <0.001 | 50.3; 26.0; 23.7 | 93.1; 91.1; 93.1 |
| 4 | -51700 | 15 | 103431 | 103524 | 103476 | 0.814 | <0.001 | 39.5; 17.0; 11.8; 31.7 | 89.4; 88.2; 90.9; 89.0 |
| **Externalizing behaviors** | | | | | | | | | |
| 1 | -34532 | 6 | 69075 | 69113 | 69094 | — | — | 100 | 100 |
| 2 | -32134 | 9 | 64285 | 64341 | 64313 | 0.822 | <0.001 | 53.8; 46.2 | 96.2; 93.1 |
| **3** | -31210 | 12 | 62444 | 62519 | 62481 | 0.815 | <0.001 | 18.5; 46.1; 35.4 | 89.0; 91.7; 92.5 |
| 4 | -30908 | 15 | 61846 | 61939 | 61892 | 0.779 | <0.001 | 35.0; 30.1; 25.0; 10.0 | 85.2; 86.5; 93.1; 86.8 |
| **Internalizing behaviors** | | | | | | | | | |
| 1 | -40840 | 6 | 81692 | 81729 | 81710 | — | — | 100 | 100 |
| 2 | -38632 | 9 | 77281 | 77337 | 77308 | 0.789 | <0.001 | 51.7; 48.3 | 94.7; 92.9 |
| **3** | -37747 | 12 | 75518 | 75592 | 75554 | 0.800 | <0.001 | 49.9; 21.8; 28.3 | 91.2; 88.4; 92.3 |
| 4 | -37500 | 15 | 75030 | 75123 | 75076 | 0.757 | <0.001 | 19.2; 10.7; 37.8; 32.3 | 89.0; 83.8; 86.1; 84.5 |
| **Thought problem** | | | | | | | | | |
| 1 | -23941 | 6 | 47894 | 47931 | 47912 | — | — | 100 | 100 |
| **2** | -22035 | 9 | 44088 | 44144 | 44115 | 0.787 | <0.001 | 64.5; 35.5 | 95.7; 91.0 |
| 3 | -21483 | 12 | 42990 | 43065 | 43027 | 0.767 | <0.001 | 42.6; 11.4; 46.1 | 89.5; 85.0; 89.9 |
| 4 | -21331 | 15 | 42691 | 42785 | 42737 | 0.729 | <0.001 | 31.0; 43.9; 4.3; 20.7 | 85.5; 85.2; 82.4; 81.8 |
| **Social problem** | | | | | | | | | |
| 1 | -23348 | 6 | 46708 | 46745 | 46726 | — | — | 100 | 100 |
| **2** | -21288 | 9 | 42594 | 42650 | 42621 | 0.811 | <0.001 | 64.6; 35.4 | 96.7; 90.5 |
| 3 | -20767 | 12 | 41558 | 41632 | 41594 | 0.779 | <0.001 | 45.9; 12.0; 42.1 | 91.2; 85.0; 90.4 |
| 4 | -20623 | 15 | 41277 | 41370 | 41322 | 0.730 | <0.001 | 5.0; 36.2; 38.5; 20.3 | 82.5; 89.2; 83.6; 80.6 |
| **Attention problem** | | | | | | | | | |
| 1 | -29777 | 6 | 59566 | 59603 | 59584 | — | — | 100 | 100 |
| **2** | -26988 | 9 | 53994 | 54050 | 54021 | 0.855 | <0.001 | 54.1; 45.9 | 95.7; 96.0 |
| 3 | -26104 | 12 | 52231 | 52306 | 52268 | 0.826 | <0.001 | 40.2; 20.3; 39.5 | 94.6; 90.5; 90.5 |
| 4 | -25820 | 15 | 51671 | 51764 | 51716 | 0.794 | <0.001 | 25.4; 33.0; 31.8; 9.8 | 86.7; 96.2; 82.2; 87.1 |
| **Anxious/depressed** | | | | | | | | | |
| 1 | -31255 | 6 | 62522 | 62559 | 62540 | — | — | 100 | 100 |
| **2** | -29127 | 9 | 58272 | 58327 | 58299 | 0.790 | <0.001 | 55.9; 44.1 | 95.7; 91.9 |
| 3 | -28352 | 12 | 56727 | 56802 | 56764 | 0.797 | <0.001 | 48.2; 34.9; 16.9 | 90.3; 93.4; 87.3 |
| 4 | -28161 | 15 | 56352 | 56445 | 56398 | 0.737 | <0.001 | 23.7; 37.8; 9.4; 29.1 | 89.7; 83.4; 86.0; 82.8 |
| **Withdrawn/depressed** | | | | | | | | | |
| 1 | -22178 | 6 | 44368 | 44405 | 44386 | — | — | 100 | 100 |
| 2 | -20374 | 9 | 40766 | 40822 | 40794 | 0.799 | <0.001 | 36.1; 63.9 | 91.9; 95.9 |
| 3 | -19955 | 12 | 39934 | 40008 | 39970 | 0.752 | <0.001 | 12.6; 38.9; 48.4 | 89.5; 87.1; 90.7 |
| 4 | -19859 | 15 | 39748 | 39841 | 39793 | 0.685 | <0.001 | 21.2; 34.1; 38.5; 6.3 | 82.1; 81.2; 81.5; 87.6 |
| **Somatic complaints** | | | | | | | | | |
| 1 | -25292 | 6 | 50596 | 50634 | 50615 | — | — | 100 | 100 |
| 2 | -23674 | 9 | 47365 | 47421 | 47392 | 0.747 | <0.001 | 43.3; 56.7 | 89.1; 95.5 |
| 3 | -23206 | 12 | 46436 | 46511 | 46473 | 0.742 | <0.001 | 49.8; 34.9; 15.3 | 86.7; 91.1; 88.3 |
| 4 | -23127 | 15 | 46285 | 46378 | 46330 | 0.686 |  | 25.3; 5.5; 45.2; 24.1 | 80.7; 74.9; 85.3; 76.9 |
| **Rule-breaking behavior** | | | | | | | | | |
| 1 | -18644 | 6 | 37300 | 37337 | 37318 | — | — | 100 | 100 |
| **2** | -16935 | 9 | 33888 | 33944 | 33916 | 0.798 | <0.001 | 68.5; 31.5 | 96.1; 91.1 |
| 3 | -16502 | 12 | 33028 | 33102 | 33064 | 0.787 | <0.001 | 32.9; 7.2; 60.0 | 81.3; 81.7; 97.5 |
| 4 | -16367 | 15 | 32764 | 32857 | 32809 | 0.752 | <0.001 | 49.9; 14.6; 33.7; 1.9 | 94.6; 81.8; 78.5; 81.9 |
| **Aggressive behavior** | | | | | | | | | |
| 1 | -31004 | 6 | 62021 | 62058 | 62039 | — | — | 100 | 100 |
| **2** | -28602 | 9 | 57223 | 57279 | 57250 | 0.826 | <0.001 | 52.8; 47.2 | 95.5; 94.2 |
| 3 | -27695 | 12 | 55414 | 55489 | 55451 | 0.822 | <0.001 | 16.7; 46.0; 37.3 | 87.6; 92.5; 93.4 |
| 4 | -27435 | 15 | 54900 | 54993 | 54945 | 0.774 | <0.001 | 27.4; 36.3;27.0; 9.3 | 84.4; 85.7; 89.7; 87.4 |

**Note:** No.: Number of classes, df: degrees of freedom, LL: Log-likelihood, AIC: Akaike information criterion, BIC: Bayesian information criterion, aBIC: Simple size adjusted BIC, LMR: Lo-Mendell-Rubin, APPA: Average posterior probability of assignment.

**Table S6**. Means and Standard Deviations for CBCL subscales across all time points, separately for each class.

|  | **Subjects, n (%)** | **Baseline** | **Year 1** | **Year 2** | **Year 3** |
| --- | --- | --- | --- | --- | --- |
| **Total problem** |  |  |  |  |  |
| Class-1 | 3823 (48.7) | 13.27 (7.87) | 12.5 (6.80) | 11.65 (6.92) | 11.86 (7.87) |
| Class-2 | 2262 (28.8) | 37.34 (19.35) | 37.73 (18.68) | 35.78 (18.86) | 36.1 (19.55) |
| Class-3 | 1764 (22.5) | 3.64 (3.21) | 3.22 (2.68) | 2.66 (2.41) | 2.83 (2.86) |
| Overall | 7849 (100.0) | 18.04 (17.48) | 17.68 (17.34) | 16.59 (16.99) | 16.82 (17.47) |
| **Externalizing behaviors** |  |  |  |  |  |
| Class-1 | 2835 (36.1) | 6.64 (6.17) | 6.49 (6.01) | 5.74 (5.87) | 5.88 (6.11) |
| Class-2 | 1620 (20.6) | 39.73 (21.34) | 39.91 (20.85) | 38.01 (20.91) | 38.14 (21.59) |
| Class-3 | 3394 (43.2) | 17.22 (11.11) | 16.42 (10.74) | 15.41 (10.57) | 15.78 (11.59) |
| Overall | 7849 (100.0) | 4.29 (5.60) | 4.11 (5.46) | 3.86 (5.40) | 3.96 (5.48) |
| **Internalizing behaviors** |  |  |  |  |  |
| Class-1 | 3759 (47.9) | 16.06 (11.87) | 15.4 (10.96) | 14.3 (10.75) | 14.56 (11.54) |
| Class-2 | 2273 (29.0) | 6.55 (6.78) | 5.79 (5.99) | 5.05 (5.73) | 5.24 (6.20) |
| Class-3 | 1817 (23.1) | 36.53 (21.34) | 37.28 (20.81) | 35.75 (20.56) | 35.97 (21.22) |
| Overall | 7849 (100.0) | 5.08 (5.44) | 5.22 (5.56) | 5.04 (5.64) | 5.31 (6.07) |
| **Thought problem** |  |  |  |  |  |
| Class-1 | 5211 (66.4) | 0.66 (0.87) | 0.61 (0.83) | 0.51 (0.77) | 0.5 (0.79) |
| Class-2 | 2634 (33.6) | 3.56 (2.60) | 3.74 (2.65) | 3.38 (2.51) | 3.28 (2.55) |
| Overall | 7845 (100.0) | 1.63 (2.15) | 1.66 (2.23) | 1.47 (2.08) | 1.44 (2.08) |
| **Social problem** |  |  |  |  |  |
| Class-1 | 5147 (65.6) | 10.4 (8.95) | 10 (8.59) | 9.29 (8.60) | 9.65 (9.35) |
| Class-2 | 2697 (34.4) | 32.63 (20.28) | 32.35 (20.14) | 30.49 (20.09) | 30.49 (20.89) |
| Overall | 7844 (100.0) | 1.59 (2.24) | 1.49 (2.15) | 1.32 (2.07) | 1.22 (1.96) |
| **Attention problem** |  |  |  |  |  |
| Class-1 | 3973 (50.6) | 8.86 (8.18) | 8.4 (7.70) | 7.65 (7.55) | 7.96 (8.42) |
| Class-2 | 3873 (49.3) | 27.47 (19.36) | 27.22 (19.21) | 25.76 (18.99) | 25.9 (19.56) |
| Overall | 7846 (100.0) | 2.97 (3.43) | 2.9 (3.43) | 2.77 (3.32) | 2.86 (3.36) |
| **Anxious/depressed** |  |  |  |  |  |
| Class-1 | 4249 (54.1) | 10.21 (9.87) | 9.46 (9.15) | 8.6 (8.82) | 8.72 (9.23) |
| Class-2 | 3596 (45.8) | 27.3 (19.83) | 27.4 (19.56) | 26.01 (19.33) | 26.39 (19.91) |
| Overall | 7845 (100.0) | 2.54 (3.04) | 2.59 (3.06) | 2.36 (2.98) | 2.39 (3.11) |
| **Withdrawn/depressed** |  |  |  |  |  |
| Class-1 | 2875 (36.6) | 29.26 (20.57) | 29.51 (20.35) | 28.35 (19.95) | 28.94 (20.47) |
| Class-2 | 4971 (63.3) | 11.56 (11.08) | 10.85 (10.36) | 9.78 (9.96) | 9.81 (10.27) |
| Overall | 7846 (100.0) | 1.01 (1.66) | 1.13 (1.79) | 1.27 (1.97) | 1.52 (2.21) |
| **Somatic complaints** |  |  |  |  |  |
| Class-1 | 3204 (40.8) | 26.78 (20.17) | 26.57 (20.02) | 25.45 (19.64) | 25.44 (20.20) |
| Class-2 | 4642 (59.1) | 12.02 (12.11) | 11.56 (11.81) | 10.47 (11.4) | 10.87 (12.15) |
| Overall | 7846 (100.0) | 1.53 (1.95) | 1.5 (1.97) | 1.41 (1.92) | 1.4 (1.98) |
| **Rule-breaking behavior** |  |  |  |  |  |
| Class-1 | 5326 (67.9) | 11.48 (10.49) | 11 (10.06) | 10.18 (9.85) | 10.41 (10.53) |
| Class-2 | 2518 (32.1) | 31.93 (20.86) | 31.82 (20.69) | 30.12 (20.59) | 30.36 (21.13) |
| Overall | 7844 (100.0) | 1.12 (1.73) | 1.09 (1.74) | 1.03 (1.78) | 1.07 (1.85) |
| **Aggressive behavior** |  |  |  |  |  |
| Class-1 | 4045 (51.5) | 8.7 (7.79) | 8.31 (7.38) | 7.51 (7.33) | 7.6 (7.47) |
| Class-2 | 3798 (48.4) | 27.99 (19.35) | 27.67 (19.23) | 26.25 (18.91) | 26.63 (19.61) |
| Overall | 7843 (100.0) | 3.17 (4.20) | 3.02 (4.07) | 2.83 (3.96) | 2.89 (3.97) |

**Table S7**. Means and Standard Deviations for CBCL Subscales Across All Time Points, Separately for Each Class in Males.

|  | **Subjects, n (%)** | **Baseline** | **Year 1** | **Year 2** | **Year 3** |
| --- | --- | --- | --- | --- | --- |
| **Total problem** |  |  |  |  |  |
| Class-1 | 1276 (30.7) | 39.43 (19.8) | 39.16 (18.64) | 37.14 (19.1) | 36.47 (19.83) |
| Class-2 | 1981 (47.6) | 14.2 (8.25) | 13.24 (7.13) | 12.17 (7.03) | 11.87 (7.54) |
| Class-3 | 904 (21.7) | 3.98 (3.47) | 3.37 (2.82) | 2.82 (2.5) | 2.84 (2.8) |
| Overall | 4161 (100.00) | 19.72 (18.51) | 19.04 (18.05) | 17.8 (17.76) | 17.45 (17.93) |
| **Externalizing behaviors** |  |  |  |  |  |
| Class-1 | 1837 (44.1) | 3.96 (2.93) | 3.6 (2.65) | 3.32 (2.55) | 3.4 (2.70) |
| Class-2 | 948 (22.8) | 12.78 (7.49) | 12.45 (6.87) | 12.05 (7.03) | 12.03 (7.40) |
| Class-3 | 1376 (33.1) | 0.67 (1.01) | 0.54 (0.84) | 0.42 (0.74) | 0.48 (0.79) |
| Overall | 4161 (100.00) | 4.88 (6.11) | 4.61 (5.83) | 4.35 (5.78) | 4.40 (5.89) |
| **Internalizing behaviors** |  |  |  |  |  |
| Class-1 | 1894 (45.5) | 4.27 (2.99) | 4.17 (2.72) | 3.82 (2.54) | 3.84 (2.91) |
| Class-2 | 1249 (30.0) | 1.08 (1.28) | 0.91 (1.08) | 0.73 (0.99) | 0.78 (1.09) |
| Class-3 | 1018 (24.5) | 11.57 (6.4) | 12.14 (6.03) | 11.72 (6.3) | 11.49 (6.72) |
| Overall | 4161 (100.0) | 5.10 (5.48) | 5.14 (5.51) | 4.83 (5.48) | 4.79 (5.61) |
| **Thought problem** |  |  |  |  |  |
| Class-1 | 2711 (65.2) | 0.77 (0.98) | 0.66 (0.85) | 0.55 (0.79) | 0.49 (0.75) |
| Class-2 | 1447 (34.8) | 3.82 (2.75) | 3.97 (2.69) | 3.6 (2.61) | 3.41 (2.59) |
| Overall | 4158(100.00) | 1.83 (2.32) | 1.81 (2.34) | 1.61 (2.21) | 1.50 (2.15) |
| **Social problem** |  |  |  |  |  |
| Class-1 | 1453 (34.9) | 3.84 (2.72) | 3.56 (2.56) | 3.27 (2.63) | 2.91 (2.55) |
| Class-2 | 2705 (65.1) | 0.54 (0.81) | 0.46 (0.74) | 0.35 (0.64) | 0.32 (0.63) |
| Overall | 4158 (100.00) | 1.69 (2.34) | 1.55 (2.20) | 1.37 (2.15) | 1.23 (2.01) |
| **Attention problem** |  |  |  |  |  |
| Class-1 | 2200 (52.9) | 5.75 (3.55) | 5.73 (3.54) | 5.56 (3.45) | 5.53 (3.47) |
| Class-2 | 1959 (47.1) | 0.90 (1.3) | 0.73 (1.05) | 0.68 (1.01) | 0.75 (1.11) |
| Overall | 4159 (100.0) | 3.47 (3.65) | 3.37 (3.66) | 3.26 (3.56) | 3.28 (3.55) |
| **Anxious/depressed** |  |  |  |  |  |
| Class-1 | 1836 (44.1) | 4.70 (3.32) | 4.81 (3.25) | 4.28 (3.19) | 4.11 (3.28) |
| Class-2 | 2323 (55.8) | 0.83 (1.10) | 0.76 (1.00) | 0.61 (0.91) | 0.59 (0.91) |
| Overall | 4159 (100.0) | 2.54 (3.04) | 2.55 (3.04) | 2.23 (2.88) | 2.15 (2.88) |
| **Withdrawn/depressed** |  |  |  |  |  |
| Class-1 | 2616 (62.9) | 0.30 (0.6) | 0.28 (0.55) | 0.30 (0.59) | 0.38 (0.66) |
| Class-2 | 1543 (37.1) | 2.46 (2.16) | 2.66 (2.22) | 2.90 (2.35) | 3.14 (2.42) |
| Overall | 4159 (100.0) | 1.10 (1.75) | 1.16 (1.83) | 1.27 (1.96) | 1.40 (2.06) |
| **Somatic complaints** |  |  |  |  |  |
| Class-1 | 1650 (39.7) | 2.87 (2.27) | 2.92 (2.25) | 2.81 (2.14) | 2.50 (2.37) |
| Class-2 | 2509 (60.3) | 0.53 (0.81) | 0.45 (0.72) | 0.36 (0.62) | 0.42 (0.76) |
| Overall | 4159(100.0) | 1.46 (1.93) | 1.43 (1.94) | 1.33 (1.87) | 1.24 (1.90) |
| **Rule-breaking behavior** |  |  |  |  |  |
| Class-1 | 2602 (62.5) | 0.42 (0.71) | 0.35 (0.63) | 0.28 (0.58) | 0.30 (0.59) |
| Class-2 | 1556 (37.4) | 2.86 (2.28) | 2.85 (2.15) | 2.75 (2.34) | 2.84 (2.43) |
| Overall | 4158(100.0) | 1.33 (1.91) | 1.29 (1.85) | 1.21 (1.92) | 1.25 (1.98) |
| **Aggressive behavior** |  |  |  |  |  |
| Class-1 | 2155 (51.8) | 0.89 (1.26) | 0.72 (1.05) | 0.64 (1.00) | 0.72 (1.10) |
| Class-2 | 2002 (48.1) | 6.41 (5.06) | 6.12 (4.76) | 5.85 (4.69) | 5.77 (4.81) |
| Overall | 4157 (100.0) | 3.55 (4.56) | 3.32 (4.33) | 3.14 (4.23) | 3.15 (4.26) |

**Table S8**. Means and Standard Deviations for CBCL Subscales Across All Time Points, Separately for Each Class in Females.

|  | **Subjects, n (%)** | **Baseline** | **Year 1** | **Year 2** | **Year 3** |
| --- | --- | --- | --- | --- | --- |
| **Total problem** |  |  |  |  |  |
| Class-1 | 1854 (50.3) | 12.44 (7.45) | 11.93 (6.54) | 11.25 (6.84) | 12.05 (8.28) |
| Class-2 | 960 (26.0) | 35.00 (18.45) | 36.16 (18.69) | 34.40 (18.49) | 35.98 (19.3) |
| Class-3 | 874 (23.7) | 3.35 (2.93) | 3.10 (2.56) | 2.57 (2.32) | 2.88 (2.97) |
| Overall | 3688 (100.0) | 16.16 (16.02) | 16.15 (16.36) | 15.22 (15.98) | 16.11 (16.91) |
| **Externalizing behaviors** |  |  |  |  |  |
| Class-1 | 681 (18.5) | 10.75 (6.32) | 11.1 (6.53) | 10.44 (6.79) | 10.34 (6.81) |
| Class-2 | 1700 (46.1) | 3.21 (2.58) | 2.95 (2.18) | 2.77 (2.32) | 3.04 (2.73) |
| Class-3 | 1307 (35.4) | 0.46 (0.79) | 0.38 (0.67) | 0.32 (0.63) | 0.42 (0.79) |
| Overall | 3688(100.0) | 3.63 (4.87) | 3.55 (4.95) | 3.32 (4.88) | 3.46 (4.93) |
| **Internalizing behaviors** |  |  |  |  |  |
| Class-1 | 1842 (49.9) | 4.34 (2.95) | 4.41 (2.78) | 4.38 (2.88) | 5.05 (3.69) |
| Class-2 | 803 (21.8) | 11.89 (6.6) | 12.86 (6.52) | 12.97 (6.92) | 14.13 (7.80) |
| Class-3 | 1043 (28.3) | 1.05 (1.27) | 1.06 (1.18) | 0.91 (1.11) | 1.05 (1.31) |
| Overall | 3688 (100.0) | 5.05 (5.41) | 5.30 (5.61) | 5.27 (5.80) | 5.90 (6.50) |
| **Thought problem** |  |  |  |  |  |
| Class-1 | 2378 (64.5) | 0.54 (0.77) | 0.50 (0.74) | 0.41 (0.68) | 0.46 (0.76) |
| Class-2 | 1309 (35.5) | 2.99 (2.35) | 3.28 (2.52) | 2.97 (2.30) | 3.00 (2.42) |
| Overall | 3687(100.0) | 1.41 (1.93) | 1.49 (2.09) | 1.32 (1.92) | 1.36 (1.98) |
| **Social problem** |  |  |  |  |  |
| Class-1 | 2380 (64.5) | 0.50 (0.80) | 0.4 (0.64) | 0.34 (0.62) | 0.36 (0.66) |
| Class-2 | 1306 (35.4) | 3.24 (2.55) | 3.29 (2.48) | 2.98 (2.40) | 2.73 (2.37) |
| Overall | 3686(100.00) | 1.47 (2.10) | 1.42 (2.09) | 1.27 (1.97) | 1.20 (1.89) |
| **Attention problem** |  |  |  |  |  |
| Class-1 | 1993 (54.1) | 0.57 (0.93) | 0.5 (0.8) | 0.46 (0.78) | 0.55 (0.91) |
| Class-2 | 1694 (45.9) | 4.56 (3.3) | 4.55 (3.28) | 4.27 (3.19) | 4.54 (3.29) |
| Overall | 3687 (100.0) | 2.40 (3.07) | 2.36 (3.06) | 2.21 (2.93) | 2.38 (3.06) |
| **Anxious/depressed** |  |  |  |  |  |
| Class-1 | 2059 (55.8) | 0.89 (1.1) | 0.92 (1.14) | 0.77 (1.02) | 0.87 (1.17) |
| Class-2 | 1627 (44.1) | 4.62 (3.42) | 4.78 (3.4) | 4.68 (3.39) | 4.95 (3.75) |
| Overall | 3686 (100.0) | 2.54 (3.04) | 2.63 (3.09) | 2.50 (3.08) | 2.67 (3.33) |
| **Withdrawn/depressed** |  |  |  |  |  |
| Class-1 | 1332 (36.1) | 2.05 (2.02) | 2.48 (2.18) | 2.97 (2.40) | 3.73 (2.78) |
| Class-2 | 2355 (63.9) | 0.26 (0.56) | 0.31 (0.59) | 0.32 (0.60) | 0.47 (0.77) |
| Overall | 3687 (100.0) | 0.91 (1.55) | 1.10 (1.74) | 1.27 (1.98) | 1.64 (2.37) |
| **Somatic complaints** |  |  |  |  |  |
| Class-1 | 1598 (43.3) | 3.00 (2.15) | 2.98 (2.21) | 2.85 (2.24) | 2.95 (2.34) |
| Class-2 | 2089 (56.6) | 0.54 (0.82) | 0.51 (0.79) | 0.46 (0.74) | 0.53 (0.85) |
| Overall | 3687 (100.0) | 1.61 (1.97) | 1.58 (1.99) | 1.50 (1.97) | 1.58 (2.05) |
| **Rule-breaking behavior** |  |  |  |  |  |
| Class-1 | 2525 (68.5%) | 0.27 (0.55) | 0.21 (0.48) | 0.19 (0.46) | 0.22 (0.52) |
| Class-2 | 1161 (31.5%) | 2.23 (1.91) | 2.29 (2.08) | 2.25 (2.16) | 2.3 (2.28) |
| Overall | 3686 (100.00) | 0.89 (1.48) | 0.87 (1.57) | 0.84 (1.59) | 0.88 (1.66) |
| **Aggressive behavior** |  |  |  |  |  |
| Class-1 | 1947 (52.8) | 0.61 (0.96) | 0.55 (0.88) | 0.52 (0.89) | 0.63 (1.02) |
| Class-2 | 1739 (47.2) | 5.13 (4.17) | 5.06 (4.2) | 4.67 (4.20) | 4.78 (4.16) |
| Overall | 3686 (100.0) | 2.74 (3.71) | 2.68 (3.71) | 2.48 (3.61) | 2.58 (3.61) |
